# Physiologically-relevant light exposure and light behaviour in Switzerland and Malaysia

**DOI:** 10.1038/s41370-025-00825-8

**Published:** 2025-12-03

**Authors:** Anna M. Biller, Johannes Zauner, Christian Cajochen, Marisa A. Gerle, Vineetha Kalavally, Anas Mohamed, Lukas Rottländer, Ming-Yi Seah, Oliver Stefani, Manuel Spitschan

**Affiliations:** 1 Department of Health and Sport Sciences, Technical University of Munich, TUM School of Medicine and Health, Chronobiology & Health, Munich, Germany; 2 Translational Sensory & Circadian Neuroscience, Max Planck Institute for Biological Cybernetics, Tübingen, Germany; 3 Centre for Chronobiology, University Psychiatric Clinics Basel (UPK), Basel, Switzerland; 4 Department of Electrical and Computer Systems Engineering, Intelligent Lighting Laboratory, School of Engineering, Monash University Malaysia, Jalan Lagoon Selatan, 47500 Bandar Sunway, Selangor Malaysia; 5 Lucerne School of Engineering and Architecture, Lucerne University of Applied Sciences and Arts, Horw, Switzerland; 6 TUM Institute for Advanced Study (TUM-IAS), Technical University of Munich, Garching, Germany; 7 TUMCREATE Ltd., Singapore, Singapore

**Keywords:** Light, Lighting, Individual differences, Cultural characteristics, Wearable technology, Photobiology

## Abstract

**Background:**

Light synchronises the internal clock with the external light-dark cycle. Keeping this alignment benefits health and prevents diseases. Quantifying light exposure is, therefore, vital for effective prevention.

**Objective:**

Since light exposure depends on photoperiod, culture, and behaviour, we investigated objective light exposure and individual light-related behaviour in Switzerland and Malaysia.

**Methods:**

In this observational field study, participants (*N* = 39) wore a calibrated melanopic light logger at chest level for 30 consecutive days. At baseline and study end, the Pittsburgh Sleep Quality Index was assessed, and every 3 to 4 days, the Light Exposure Behaviour Assessment (LEBA) was filled.

**Results:**

Our pre-registered analyses reveal that participants in Switzerland experienced brighter days (+3.16 times the average mel EDI) and spent more time (x1.9 times the duration) in daylight levels per hour of daylight, had ~1.5 h later bright light exposure in the afternoon, and stayed over 1 h longer in dim light conditions before bedtime. LEBA scores did not differ between Malaysia and Switzerland, and LEBA items were stable over time. LEBA items also correlated with objective light exposure variables in Switzerland but not Malaysia, with a medium effect size (range of absolute *r* = 0.32–0.48).

**Significance:**

Our results highlight cultural and geographical differences in light exposure. We showed that subjective assessment of light-exposure behaviour can be related to actual light exposure and is ecologically informative, but this varies by culture.

**Impact:**

- Light is a key environmental factor shaping human health, yet real-world exposure varies widely across geography and culture. In this study, we compared physiologically-relevant light exposure and self-reported light-related behaviour between Switzerland and Malaysia over a 30 day period using wearable sensors and questionnaires. We found that participants in Switzerland experienced brighter days and darker evenings than those in Malaysia, even after accounting for differences in photoperiod length. These differences reflect how climate, culture, and behaviour influence light exposure patterns. Our findings highlight the need to consider cultural and environmental context when developing recommendations and interventions to promote healthy light exposure in everyday life.

## Introduction

Light is the main entrainment stimulus (zeitgeber) for synchronising human circadian rhythms to the natural light-dark rhythm of daylight given by Earth’s rotation (e.g. [1–4]). A predictable and strong zeitgeber is key for a functioning sleep-wake rhythm and for human physiology to best deal with daily demands and stressors (e.g. [5–8]). With the broad adoption of electrical light in the 19th and 20th century, the daily pattern of light exposure has rapidly altered from the robust light-dark cycle characterised by bright days and dark nights to a less predictable one, resulting from a potentially incalculable combination of artificial lights in the built environment and behavioural interactions with natural light and electrical light sources [9]. Additionally, bright days become dimmer due to a switch from daylight to weaker electrical sources indoors, while dark nights are brightened by electrical light (artificial light at night; ALAN). Factors that drastically influence natural exposure to daylight on a societal level are, to a large degree, the level of industrialisation and access to electricity [10], and consequently, working hours such as shift work [11].

Disrupting the entrainment of endogenous circadian rhythms by shifted or disordered light exposure can have serious health consequences, such as disrupted sleep, shift work disorder, metabolic disorders, cardiovascular diseases, mood disorders, impaired immune system, or certain cancers (e.g. Ansu Baidoo and Knutson [12]; Fishbein et al. [13]; Lunn et al. [14]; Neves et al. [15]; Roenneberg and Merrow [4]). Recognising these negative consequences has led to a recent consensus statement on the optimal light exposure levels to best support physiology, sleep, and wakefulness in healthy adults [16]. This consensus statement was strengthened through a concerted call to apply these recommendations in policy-making, lighting design and preventative measures for public and occupational health [17], leading to evidence-based statements for use in public health contexts [18, 19]. Analysing light exposure is, therefore, the necessary basis for restoring healthy, strong, and reliable circadian rhythmicity to both mitigate the negatives and increase the benefits.

However, the current expert recommendations by Brown and colleagues, albeit a beneficial and important start, especially for public health, are based on isolated laboratory lighting conditions and do not factor in individual light *behaviour* [9]. Photic history [20–22] and an individual’s spectral diet [23]—the timing and spectral composition of prior light exposure—also play a role in subsequent sensitivity to light. Recommendations for healthier light exposure thus also need to factor in individual light exposure during day and night and how they interact with light sources. At present, there is minimal data about real-world light exposure and how much humans deviate from these target light levels. Previous studies often only sampled for a limited amount of time (e.g. several days to 1 or 2 weeks) and used wrist-based light loggers from actimeters (e.g. Didikoglu et al. [24]) or devices worn at spectacle frames [25], the latter being valuable but limited in usability for more extended wear periods. Novel light logging devices at physiologically more relevant wearing positions (e.g. chest, eye level) are now available, thus quantifying ecological light exposure over long periods. One such device is the SPECCY light logger [26], a chest-worn wearable light logger that outputs numerous visual and non-visual metrics of light exposure following relevant standards from the CIE (International Commission on Illumination) standards via a spectral reconstruction algorithm [27].

Since individual light exposure-related behaviours also depend on an individual’s location (e.g. photoperiod, climate, temperature), culture, and the built environment, quantifying light exposure should factor in such behavioural and structural components [9]. The Light Exposure Behaviour Assessment (LEBA) instrument [28] has recently been developed to quantify individual light-related behaviours. It categorises light exposure-related behaviours into five broad categories (factors) based on the participant’s recall of the past 4 weeks: (i) wearing blue light filters, (ii) spending time outdoors, (iii) using phones and smartwatches in bed, (iv) using light before bedtime, and (v) using light in the morning and during daytime. This questionnaire is well-suited to map objectively measured light exposure patterns to individual self-reported behaviours, but also lends itself to studying potential cultural differences in interactions with light.

This project had three objectives. The first objective was to investigate differences in objectively measured physiologically relevant light exposure light loggers at two sites that vary in culture and climate—Malaysia compared to Switzerland. A second objective was to investigate differences between the sites in subjectively measured light exposure behaviour using the LEBA questionnaire. The third objective was to examine which objectively derived light variables correlate with LEBA-derived items and composite scores and whether this depended on the study location.

Knowing more about the scope of assessment methods and cultural differences in light exposure and interactions with light can help inform future tailored and public health interventions to improve and foster healthy light exposure [9].

## Methods

The current study was preregistered [29]. We describe deviations from the preregistration document in the ‘Deviation from the preregistration’ section below.

### Research questions and hypotheses

We addressed the following research questions and hypotheses as specified in the pre-registration document:

- Are there differences in objectively measured light exposure between Switzerland and Malaysia, and if so, in which light metrics?

- **H1:** There are differences in light logger-derived light exposure intensity levels and intensity duration between Malaysia and Switzerland.
- **H0**_**1**_**:** No differences between Malaysia and Switzerland.
- **H2:** There are differences in light logger-derived timing of light exposure between Malaysia and Switzerland.
- **H0**_**2**_**:** No differences between Malaysia and Switzerland.

- Are there differences in self-reported light exposure patterns using LEBA across time or between the two sites, and if so, in which questions/scores?

- **H3:** There are differences in LEBA items and factors between Malaysia and Switzerland.
- **H0**_**3**_**:** No differences between Malaysia and Switzerland.
- **H4:** LEBA scores vary over time within participants.
- **H0**_**4**_**:** No differences in LEBA scores over time among participants.

- In general, how are light exposure and LEBA related, and are there differences in this relationship between the two sites?

- **H5:** LEBA items correlate with preselected light-logger-derived light exposure variables.
- **H0**_**5**_**:** No correlation.
- **H6:** There is a difference between Malaysia and Switzerland in how well light-logger-derived light exposure variables correlate with subjective LEBA items.
- **H0**_**6**_**:** No differences between Malaysia and Switzerland.

### Study design

**Fig. 1:**
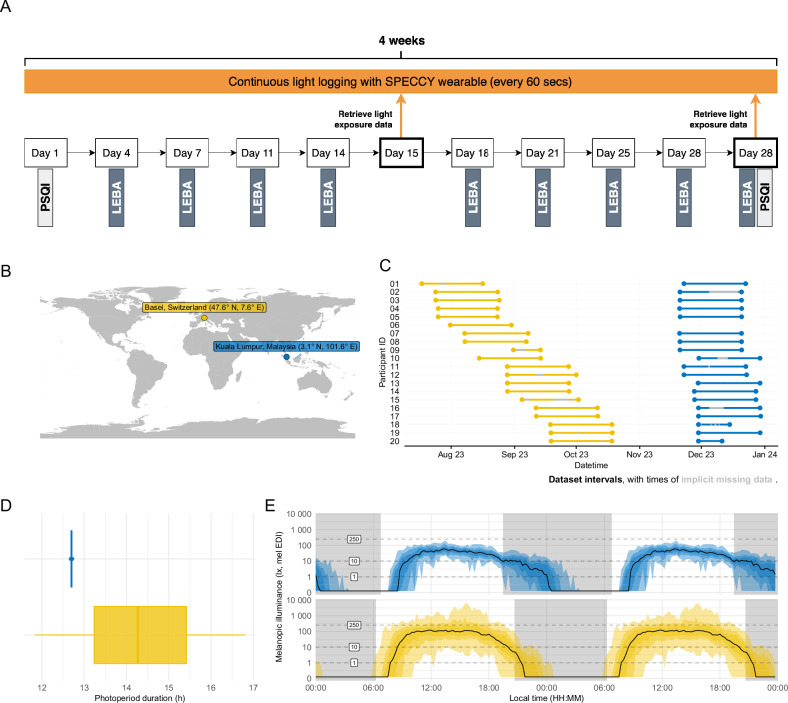
Overview of the study design. **A** Shows the study design and measurements included for both research sites. **B** Both research locations (Basel, Switzerland; Kuala Lumpur, Malaysia) shown on a world map. **C** Individual recording periods per site and participant including implicit missing data in grey. Note that participant ID 06 is missing in the Malaysian dataset due to a lost light logger. **D** Photoperiod duration in Malaysia (blue), and Switzerland (yellow). **E** Grand averages of light exposure of illuminance (melanopic EDI) for Singapore (blue) and Switzerland (yellow). Abbreviations: LEBA Light exposure behaviour assessment, mel EDI melanopic equivalent daylight illuminance, PSQI Pittsburgh Sleep Quality Index.

This observational study took place in Kuala Lumpur, Malaysia, and Basel, Switzerland, and was conducted for 1 month per participant at each site. Participants were not randomly assigned to treatment nor blinded in any way and were allowed to live their regular lives as usual. Day 0 and day 31 involved the collection and return of equipment, while days 1–30 involved the collection of light exposure and questionnaire data from the participants (Fig. 1A). Light data were collected via a neck-worn portable light logger (SPECCY). Questionnaires were deployed online via Qualtrics (Malaysia) and REDCap (Switzerland) [30, 31].

On day 0, participants collected the light logger and filled in the Pittsburgh Sleep Quality Index (PSQI) questionnaire. The light logger was worn around the neck at chest level, approximately 20 cm below the wearer’s chin, with the sensing area facing outwards towards the sight of light arriving on the wearer’s eyes. From day 1 to day 14, participants wore the light logger continuously except when exposed to high amounts of water (e.g. bath, swimming) or when the dosimeter was being charged. Participants were asked to charge the sensor daily for an hour. The participants chose this charging time themselves, but it was recommended to the participants that this charging time would remain consistent throughout the experiment and preferably at a time when they were primarily sedentary. Participants were also instructed to ensure that the sensor area of the light logger was not covered by any obstacles or objects (e.g. hands, hair, seatbelts, etc.). No specific instructions were given on light logger placement while participants were asleep. When the light logger was not worn, participants were instructed to place it so that its sensor area was blocked and could not detect any light. Participants were asked to answer the LEBA questionnaire twice a week before going to bed at night. On day 15, the light exposure data recorded by the light logger were downloaded from the device, and on day 31, participants returned the device, after which the collected light exposure data were downloaded. Data collection was completed after participants answered the PSQI questionnaire.

### Inclusion and exclusion criteria

In total, 20 (Switzerland) and 19 (Malaysia) participants were recruited to participate in the data collection experiment with the following self-reported (no standardised questionnaires) inclusion criteria:

- Age between 19 and 65 years old
- No history of drug abuse
- Caffeine consumption ≤2 cups per day
- Alcohol consumption <2 drinks per day or 14 drinks a week
- Smoke <2 cigarettes per day

### Data collection procedures

Data collection took place at the Psychiatric University Clinics (UPK), Basel, Switzerland (47.5714°N, 7.5677°E) and at Monash University, Kuala Lumpur, Malaysia (3.0650°N, 101.6009°E), but participants were allowed to roam freely (Fig. 1B). Data collection in Switzerland was carried out between July and October 2023 (summer/autumn), while data collection in Malaysia was carried out between November and December 2023 (Fig. 1C). Photoperiod change during the collection period was considerable in Switzerland, but not Malaysia (Fig. 1D). Participants were asked to stay within a 50 km radius of the respective sites.

For participants in Malaysia, applicants were asked to answer questions related to the inclusion/exclusion criteria using Google Forms. A total of 50 applicants were registered. Fourteen applicants did not meet the inclusion criteria, and the remaining applicants were either accepted, declined participation, or not contacted due to the maximum sample size of twenty being reached. One person was later excluded from the analysis due to a lost SPECCY device, leading to a final sample size of 19 participants in the Malaysian dataset. Qualtrics links to the LEBA and PSQI questionnaires were sent to the participants according to their schedules, and they would fill in the questionnaires via the link.

Participants in Switzerland were recruited through an online advertisement posted on the University’s bulletin board. The advertisement included a brief description of the study and the inclusion and exclusion criteria. Those who expressed interest in the study were contacted via email to verify their eligibility. Upon confirmation, they were provided with a detailed study information sheet. Eligible and interested applicants were scheduled to visit the lab on a Monday. Participants were briefed in person about the study and registered using Qualtrics. Participants also completed the PSQI on-site using their mobile phones and received instructions on how to use the light logger.

### Wearable light logger

A wearable light logger, SPECCY, was used for data collection in Malaysia and Switzerland. The light logger, modified from the light spectral sensor developed by Mohamed et al. [26] and developed at Monash University Malaysia’s Intelligent Lighting Laboratory, captures spectral information across the visible range between 380 and 780 nm. The Australian Photometry and Radiometry Laboratory validated SPECCY, which has an effective measuring range from 1 lux to 130,000 lux. The light logger system is constructed from three multi-channel sensors, which provide 14 optical sensing channels within the measurement range of 380 nm and 780 nm and four channels in the infra-red range. These channels capture low-resolution spectral data through proprietary software layers adjusted for sensor saturation and baseline signal calibration. Incorporated within a compact printed circuit board, the device houses the three multi-channel sensors, optimally placed for temperature consistency, an ambient temperature sensor, a microprocessor enabling Bluetooth Low Energy connectivity, 16 MB of onboard flash storage allowing for over 130,000 spectral measurements, vital indicators and controls such as LEDs, a connectivity toggle button, and a micro-USB charging port. These components are securely encased within a 3D-printed housing made of polylactic material. A cosine corrector built into the case ensures appropriate sensor directionality. The output measurements are consistent with the CIE S 026/E:2018, including melanopic equivalent daylight illuminance [27].

### Surveys

#### LEBA

The Light Exposure Behaviour Assessment instrument (LEBA; Siraji et al. [28]) was developed to examine how light exposure behaviours affect health and well-being. LEBA categorises five key behaviours: wearing blue light filtering glasses indoors and outdoors (Factor 1), spending time outdoors (Factor 2), using phones and smartwatches in bed before sleep (Factor 3), controlling and using ambient light before bedtime (Factor 4), and using light in the morning and during daytime (Factor 5). Table S1 lists detailed computations of these factors.

#### PSQI

The PSQI [32]) is a self-report questionnaire that assesses sleep quality over 1 month. It measures seven components, including sleep duration, disturbances, and daytime dysfunction, and produces a global score. A higher global score (≥5) indicates poor sleep. Table S2 lists details on the computation of components and global scores.

### Sample size calculation

No specific sample size was calculated for the study. The sample was based on convenience sampling and resource limitations. No specific stopping rule was implemented.

### Measured variables

#### SPECCY-derived variables

Light data from the SPECCY device (melanopic EDI) were analysed using the R package *LightLogR v0.9.2* [33] to perform cleaning steps, create visualisations, and calculate derived light exposure metrics. Melanopic EDI, the melanopic equivalent daylight illuminance (mel EDI) measured in lux, measures how strongly light affects the melanopsin system, which is the primary driver of the non-image-forming pathways in humans [16]. An overview of each calculated light metric is given in Table S3. The exact calculation equation for each metric is contained within the open-source code of LightLogR (https://github.com/tscnlab/LightLogR).

#### LEBA-derived variables

The Light Exposure Behaviour Assessment instrument (LEBA; Siraji et al. [28]) captures light exposure-related behaviours on a 5-point Likert-type scale ranging from 1 to 5 (1 = never; 2 = rarely; 3 = sometimes; 4 = often; 5 =  always). The score of each factor is calculated by the summation of scores of items belonging to the corresponding factor (Table S1). Respondents are requested to respond to each item retrospectively to capture their propensity for different light exposure-related behaviours. Originally, this covers the past 4 weeks, but was amended in the current study to ‘past 3–4 days’ (asked twice per week) for the Swiss site (the original framing of 4 weeks was kept for the Malaysian site). A list of all LEBA items is given in Table S5.

#### Composite scores

For the LEBA, five factors were calculated based on individual LEBA items (see Table S1). In scoring the PSQI, seven component scores are derived, each scored 0 (no difficulty) to 3 (severe difficulty). The component scores are summed to produce a global score (range 0 to 21). Higher scores indicate worse sleep quality, with a score >5 suggesting significant sleep difficulties.

### Transformations of variables and preprocessing steps

#### LEBA-derived variables

Item 4 of the LEBA questionnaire was reverse-scored for the F2 calculation (see section ‘Measured variables’ for more information on items and factors of the LEBA). LEBA scores were stored in a dummy-coded way in the Switzerland dataset (exported from RedCap), where 1 to 5 encode ‘Never’, ‘Rarely’, ‘Sometimes’, ‘Often’, and ‘Always’, respectively. Answers were directly coded in the Malaysia dataset (exported from Qualtrics). Upon import, all LEBA scores were converted to dummy-coded factors as described for the Switzerland dataset.

#### Sleep times and chronotype from PSQI variables

Items 1 through 3 of the PSQI were used to calculate sleep/wake times and chronotype. Sleep onset was derived by adding typical bedtime (item 1) and sleep latency (item 2). For sleep offset, item 3 was used directly (wake time). For chronotype, the duration of sleep was calculated (sleep offset minus sleep onset on a circular scale), halved, and added to sleep onset. The resulting metric indicates the timing of mid-sleep as a proxy for chronotype (similar to chronotype, i.e. MSFsc, derived from the Munich Chronotype Questionnaire; Roenneberg et al. [3]). As free day and workday information were not collected, no sleep correction for sleep debt (as done to calculate MSFsc from the MCTQ) during working days was applied.

#### Melanopic EDI-derived variables

The base measurement was the time series of melanopic EDI (in lux). Several summary metrics are calculated from that measure (see Table S3). The period over which these metrics are calculated varies depending on the specific research question, hypothesis, and metric. Generally, metrics were calculated per participant and day, except IS, which was calculated per participant. Whenever melanopic EDI was directly used in a statistical model, it was logarithmically transformed (base 10) [34].

#### Time-of-day

For some research questions, the distinction of daytime vs. evening or nighttime was relevant when calculating metrics, as either time of day was part of the statistical model or only a portion of the day was relevant. In those cases, metrics were calculated based on a filtered time series of melanopic EDI. These filters have three thresholds: dusk, dawn, and midnight. Dusk and dawn were calculated based on latitude, longitude (Switzerland: 47.5585°N, 7.5839°E; Malaysia: 3.0650°N, 101.6009°E), and calendar date with the *suntools* package [35], with a solar depression angle of 6°, which yields civil dusk and dawn. Dawn to dusk is considered daytime, dusk to midnight as evening, and midnight to dawn as nighttime.

#### Datetimes

Since the two sites were in different time zones, directly including the datetime variable in a statistical model or a combined visualisation was not sensible since we were interested in differences depending on local time, not real-time differences due to different time zones. When only the local clock time (not the date) was of interest, datetime was transformed into seconds from midnight local time. When the local time, including date, was of interest, datetime was transformed by overwriting the time zone of both sites to UTC, thus forcing them to operate on the same local timeline.

#### Correlating LEBA scores with light exposure metrics

Depending on the site, different time spans were used in the correlation analysis (**H5**) due to how the items in the questionnaire were framed:

For Switzerland, the LEBA covered behaviour in the current respective period (i.e. about the past 3 or 4 days), and light exposure metrics were calculated from the time spans between each LEBA questionnaire. These metrics were correlated with the respective LEBA scores. For example, LEBA scores from day 7 were correlated with light exposure metrics from days 5 to 7, as the prior LEBA questionnaire had been collected at the end of day 4. All other time spans between assessments were calculated accordingly.

For Malaysia, where the LEBA had asked about the behaviour in the past 4 weeks, light exposure metrics were calculated for the entire 4 weeks of data collection and correlated with the final round of LEBA scores collected on day 31.

An average value was calculated prior to correlation for light exposure metrics that were calculated for each day. This avoided a one-to-many comparison (one LEBA datapoint to many light exposure metric datapoints) that would have skewed significance tests of the correlation.

#### Descriptive statistics and statistical models

We also tested if light exposure and LEBA items were correlated (**RQ3**) by calculating specific light variables using the R package *LightLogR* and correlating them to the respective LEBA item. See Table S5 for an overview of which light variables were calculated for each LEBA item.

#### Exploratory analyses

We extended our analyses beyond the specified hypotheses described above in a secondary analysis. Since we expected differences between the two sites regarding objectively measured light exposure (using the SPECCY light logger; **RQ1**) and subjectively measured light exposure (using the LEBA questionnaire; **RQ2**), we also wanted to test this thoroughly in a data-driven way. To this end, we did a cross-correlation of all available light metrics in *LightLogR* for the objectively measured light exposure with all available variables and factors from the LEBA questionnaire across sites. We further looked into dependencies of light exposure and derived metrics due to age, sex, and chronotype. Lastly, we assessed the compliance with the recommendations for healthy day, evening, and night light exposure [16].

### Inference criteria

#### Check for assumptions

Appropriate model diagnostics were performed for all statistical tests to ensure linearity of (generalised) relationship, normally distributed residuals and random effects, and homoscedastic residuals. In the case of generalised additive models, the number of knots (k) was tested to ensure sufficient degrees of freedom to adequately capture the nonlinear relationships.

#### Statistical tests

Table S4 links specific statistical tests to the research questions and hypotheses. Overall, one of five statistical model types was used:

1. (Generalised) linear mixed-effect models for continuous numeric dependent variables, using the lmer() and glmer() functions in the *lme4* package version 1.1-35.5 [36]. Generally, a Gaussian error distribution was assumed for dependent variables. A Poisson error distribution was used for count data, such as the seconds above a given threshold.
2. Cumulative link mixed-effect models for ordinal dependent variables, using the clmm() function in the *ordinal* package version 2023.12-4.1 [37].
3. Generalised additive mixed-effect models for non-linear relationships, using the gam() and bam() functions in the *mgcv*package version 1.9-1 [38]
4. Correlation matrices using Spearman-rank correlation for the correlation of continuous numeric and ordinal variables. These models use the cor() function in the *stats* package version 4.4.2 in base R [39] and the rcorr() function in the *Hmisc* package version 5.2-1 [40]. For the correlation of chronotype (mid-sleep) with the mean timing of light above threshold (MLiT250), we used circular correlation with the *circular* package, version 0.5-1 [41].
5. Bootstrapping to determine 95% confidence intervals for variables. Bootstrapping was performed with base R functions such as sample() and the *tidyverse* package version 2.0.0 [42, 43].

#### Accounting for multiple testing

A false-discovery-rate correction (Benjamini-Hochberg) was applied for every hypothesis based on the number of models/tests performed within that specific hypothesis.

#### Effect size

For the statistical tests in model types 1, 2, and 3, unstandardised effect sizes are reported as a result of the final models, i.e. beta-coefficients and non-linear dependency curves. For type 4, the correlation coefficients are effect sizes.

#### Significance levels

For statistical tests in model types 1, 2, and 4, *p*-values were calculated and corrected for multiple testing. Values equal to or below 0.05 were considered significant. For type 1 and type 2, *p*-values are calculated through a likelihood ratio test of models with and without a parameter in question. For type 3, model selection/significance was based on Akaike’s Information Criterion (AIC), where the more complex model was preferred if their AIC was two or more below the less complex model. For type 4, *p*-values were calculated through the asymptotic t-approximation [44]. For type 5, 95% confidence intervals were calculated based on 10^4^ bootstrapping samples. If a standard deviation of 0 was not within the confidence interval, the item variance over time was considered significant.

#### Photoperiod correction

Figure 1D shows the differences in photoperiod between the two sites during data collection. The photoperiod, as measured from civil dawn to civil dusk, was, on average, 12.7 h in Malaysia during data collection, while it was 14.4 h in Switzerland. Furthermore, while there is a negligible difference in photoperiod within the Malaysia site, there is a strong variation within the Switzerland site due to higher latitude and a longer data collection period (not per participant but for overall data collection). To account for this, metrics in H1 were corrected by their respective periods: times above or below a given threshold per day were divided by the (photo)period relevant for the metric. The resulting metrics should be interpreted such that per hour of photoperiod, *x* seconds were spent above/below the threshold. Similarly, this correction was applied for time metrics covering the evening, which were divided by the evening length (dusk until midnight).

## Data exclusion, outlier handling and awareness checks

### Exclusion criteria for data

Measurement data were excluded if their values lay outside the measurement equipment’s plausible thresholds (≥130,000 lux for photopic illuminance). No awareness checks were implemented during data collection, and outlier handling was not necessary after viewing the data.

### Missing data

The missing data percentages (implicit missing data) and percentage data where the recorded photopic illuminance fell outside the light logger’s effective measuring range between 1 lux and 130,000 lux (explicit missing data) were recorded and analysed. Values above the upper threshold were few (one data point for Malaysia and eight data points for Switzerland) and negligible relative to the amount of available data. Values below 1 lux were regular and expected, as this value indicates darkness. Values below 1 lux were converted to 0.1 lux, which allowed for their inclusion in logarithmically transformed models [34].

All implicit missing data were made explicit in the analysis, i.e. gaps in the time series were filled with the respective date, and corresponding measurement values were set to NA. Participant days with more than a fixed threshold of 6 h of missing data per day were excluded from further analysis. The threshold was determined by a sensitivity analysis using three randomly chosen light exposure metrics and three randomly selected participants without missing data. The threshold was set so that the average of each metric stayed within a 95% confidence interval based on their original variation (mean ± 2SE) and 10^4^ resamples for each threshold. This ensured a threshold that did not significantly affect metric calculation based on the actual data (see Fig. S1). While the threshold may seem rather large, it exemplifies that for a sufficiently long time series, i.e. 30 days in this case, the chosen light exposure metrics (IV, last timing below 10 lux, MP ratio) are rather robust to randomly missing data, even for larger time frames within a day. For an overview of missing data, see Table S6.

### Deviations from the preregistration

In research question 1, H2 (*‘There are differences in light logger-derived timing of light exposure between Malaysia and Switzerland’)* of the preregistration document specified an additive model to describe light exposure patterns within participants and between sites. This approach led to the conclusion that the light exposure patterns were dominantly driven by individual patterns rather than an overarching pattern by site (see *Results*). To explore differences by site, another model that only included random intercepts by participant (rather than random smooths by participant) was constructed. Both models are part of the analysis documentation in the Supplementary Materials (Section 5.2.2.4), and their results will be discussed according to their respective relevance.

The preregistration document specified a generalised mixed model approach to test H6 (‘*There are differences between Malaysia and Switzerland on how well light-logger derived light exposure variables correlate with subjective LEBA items*’)*.* As the specified formula did not lead to the stated number of models, i.e. 23 + 5, and also did not provide an overall overview of whether selected light exposure metrics correlate with LEBA items and factors, we provide here an alternative approach by analysing whether there are significant differences in correlation coefficients between the sites. In this approach, a linear model is used, and the number of models equals the number specified in the preregistration. The output is also more suitable for answering the hypothesis. The stricter analysis is still included in section 5.4.2. of the analysis documentation (linked in Supplementary material).

We also had to correct H0_4,_ which originally stated ‘*No differences between Malaysia and Switzerland*’. However, this comparison is not sensible given that we were investigating the LEBA scores within the Malaysian dataset over time instead of investigating if these scores differed over time, dependent on the research site. The updated H0_4_ is thus: ‘*No differences in LEBA scores over time within participants*’, as already stated above at the beginning of the Methods section.

## Results

### Participant characteristics

The sample in Basel, Switzerland, consisted of five male and 15 female participants (age 30.35 ± 9.7 years old), and all participants completed their data collection successfully. In Malaysia, 8 males and 11 females (age 24 ± 6.7 years old) completed data collection successfully (Fig. S3), and one participant failed to complete the experiment due to the loss of the light logger. Age differed significantly between sites *(p* = 0.03), but there was no significant dependency of age on sex (*p* = 0.97). Sex did not differ significantly by site (*p* = 0.32), nor was there a significant difference in the number of male vs. female participants overall (*p* = 0.98). PSQI scores were comparable between sites, both on day 1 and day 31, and did not differ significantly (Tables S13, S14).

Timing of mid-sleep derived from the PSQI (as a proxy for chronotype) was 4:57 a.m. ±1.4 h in Malaysia, and 3:45 a.m. ±0.8 h in Switzerland. The difference is significant (*p* = 0.003; see Fig. S5). This behavioral difference of 1.2 h in mid-sleep compares to a 0.37 h solar difference in noon timing (i.e. photoperiod centre).

### Individual differences in light exposure

**Fig. 2:**
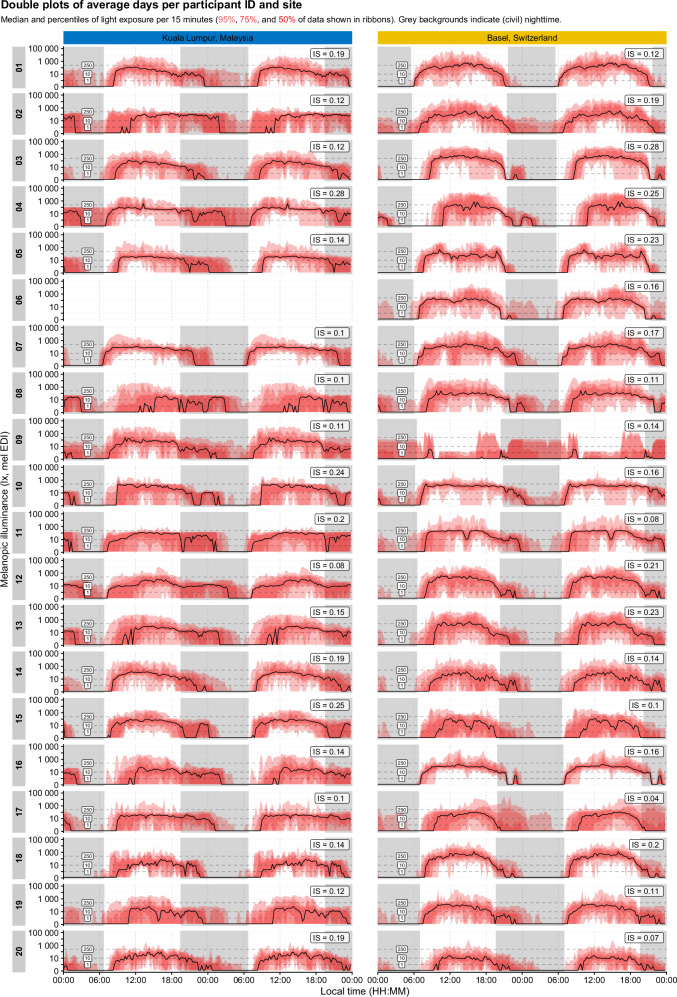
Double plots of average light exposure over participants across both research sites. Median and percentiles of light exposure measured in illuminance (lux, melanopic EDI per 15 min (95%, 75%, and 50% of data shown in ribbons). Grey background indicates civil nighttime. Abbreviations: IS Interdaily stability. Melanopic EDI Melanopic equivalent daylight illuminance.

Figure 2 shows individual light profiles from both sites averaged across the recording period. While the results below go into detail about condensed quantifiers of light exposure metrics, individual patterns lead to valuable insights into the wide breadth of light exposure patterns and their variation. To highlight just a few, some participants, such as ID 16 or 18 in the Swiss dataset, show very *small deviations* from their daily light exposure patterns as indicated by narrow 95-, 75-, and 50-percentile ribbons. On the other hand, participants 15 and 17 in the Swiss dataset or participants 08, 13 or 16 in the Malaysian dataset show *strong deviations* from their median daily patterns. Interestingly, this is somewhat captured by the respective IS measures, but not as much as expected. In general, the IS values are also rather small (all <0.3), indicating strong variation between days (higher numbers mean more stability).

Furthermore, the data also show a degree of within-day variation: while participant 08 in Malaysia shows a clear tendency for predominant nighttime exposure, there are regular ‘daytime patterns’ visible in the 95- and 75-percentile ribbons. Participant 17 in Switzerland, on the other hand, has a clear daytime exposure profile but with regular evening and nighttime extensions and variations.

### Participants in Switzerland spent more time in daylight, experienced brighter days and stayed longer in recommended light levels during the day compared to participants in Malaysia

Figure 1E shows the grand average of light exposure profiles for the site in Switzerland (yellow line) and the site in Malaysia (blue line). The model summary (Table S7) shows that participants in Switzerland spent significantly more time above threshold per hour of daylight for 250 lux (TAT250) and 1000 lux (TAT1000) than participants in Malaysia, i.e. x1.78 and x1.94, respectively (x1.77 over 250 lux melanopic EDI during daytime hours, TATd250). The second model summary (Table S9) also shows that the 10 brightest hours (M10m) of participants in Switzerland are significantly brighter than for participants in Malaysia, i.e. 661 lux and 229 lux, respectively. The frequency of crossing 250 lux is about twice as high for participants in Switzerland compared to Malaysia (64 absolute times compared to 36 times, respectively). Lastly, participants in Switzerland experienced light levels of above 250 lux melanopic EDI about 1.5 h longer across the day, compared to Malaysia (19:09 vs 17:41 respectively, Table S9) and about 2.5 times higher melanopic EDI illuminance levels daily (437 lux vs 170 lux respectively) as demonstrated in the interaction of site and time of day (Table S8).

### Participants in Switzerland experienced earlier dim light exposure and darker evenings

Participants’ last time above 10 lux after dusk (LLiT10) in Switzerland was about 1 h earlier than participants in Malaysia (22:05 vs 23:16, respectively). There was no difference (*p* < 0.9) in time below threshold of 10 lux during the evening (TBTe10) between sites (Table S7). Exploring the interaction of site and time of day (day vs evening) in illumination levels revealed an average for participants in Switzerland of 3.5 lux during evening hours and a 5-fold increase (17.6 lux) for participants in Malaysia (Table S8).

### Participants in Switzerland followed recommendations for healthy light exposure about half of the time, in Malaysia, about 40% of the time

Using PSQI-derived typical sleep and wake times to assess whether participants followed the Brown et al. [16] recommendations for healthy light exposure revealed a marked difference between both the categories and sites. These align well, however, with the results above regarding daytime and evening comparisons between sites. The lowest level of compliance to the recommendations was for daytime levels (wake until 3 h prior to sleep), where participants in Malaysia only managed to reach 250 lx melanopic EDI 10% of the time, compared to 26% in Switzerland. This increased markedly in the evening (last 3 h before sleep), where Malaysian participants stayed at or below 10 lx melanopic EDI 64% of the time, compared to 78% in Switzerland. Better still were nights (sleep hours), with 84% of Malaysian participants at or below 1 lx melanopic EDI, compared to 92% in Switzerland. Overall, participants in Malaysia followed the recommendations 39% of the time, compared to 54% in Switzerland (see Table 1). A more granular depiction of when participants followed the recommendations can be found in Fig. S6.

**Table 1:** Compliance with Brown recommendations for healthy light exposure.

	Day	Evening	Night	Overall
Malaysia	10%	64%	84%	39%
Switzerland	26%	78%	92%	54%

**Day** includes the time from wake until evening (recommendation ≥250 lx mel EDI).

**Evening** starts 3 h prior to sleep until sleep onset (recommendation ≤10 lx mel EDI).

**Night** covers the sleep times (recommendation ≤1 lx mel EDI).

**Overall** indicates the time-weighted average compliance.

Data covers all valid measurement periods for all participants. Daily values for *day*, *evening*, and *night* were averaged per participant and then per site, i.e. these represent an average participant-*day/evening/night* per site. *Overall* values represent time-weighted averages of *day*, *evening*, and *night*, i.e. values represent an average (full) participant-day per site.

### Chronotype relates to mean timing of light in Malaysia, but not Switzerland

**Fig. 3:**
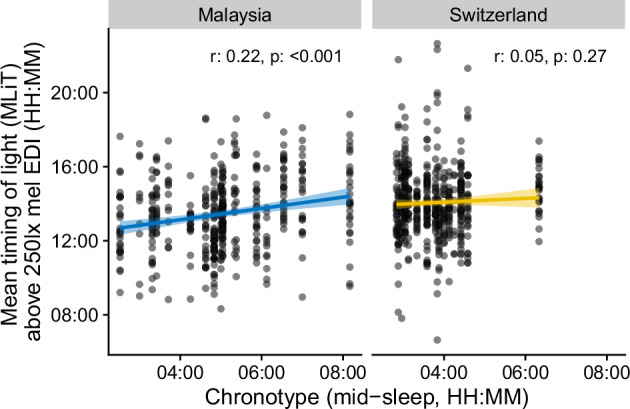
Chronotype influence on light exposure timing. Dependency of the mean timing of light above 250 lx melanopic EDI (MLiT_250_) against chronotype (mid-sleep) for each site. Dots indicate value pairs, the line and bands a linear model of the dependency. Correlation values (*r*-values) at the top of each panel show the (circular) correlation coefficient of the dependency, including its p-value.

We found a slight correlation of mean timing of light above 250 lx (MLiT_250_) with chronotype (mid-sleep) in Malaysia (*r* = 0.22, *p* < 0.001), but not in Switzerland (*r* = 0.05, *p* = 0.27; see Fig. 3). In a mixed-model framework, the overall dependency of MLiT_250_ on chronotype is significant (*p* = 0.01), but the site-specific interaction is not supported (*p* = 0.35). Thus, there is an overall prediction of 0.22 h increase in MLiT_250_ per hour increase in mid-sleep.

### Modelled light exposure patterns vary between sites, with strong differences between individuals

**Fig. 4:**
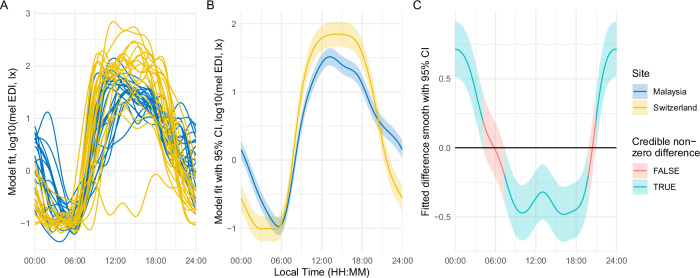
Model fits of light exposure differences between Malaysia and Switzerland. **A** Individual model fits per ID and research site for melanopic EDI (lux). **B** Model fits per research site with 95% confidence interval. **C** Fitted difference smooths between research sites with 95% confidence interval and an indication of significant differences per time. Abbreviations: CI confidence interval, mel EDI melanopic equivalent daylight illuminance.

Figure 4 shows model results when estimating light exposure patterns, with Fig. 4A showing individual patterns, Fig. 4B patterns by site, and Fig. 4C differences between the sites. While each site shows a characteristic pattern (Fig. 4B), these are composed of strongly varied individual patterns within each site (Fig. 4A). In fact, when modelling each participant’s pattern, an overall site-specific pattern does not significantly increase the model’s explanatory power. This is indicative of strong differences between participants within each site. Overall, the light exposure patterns are consistent with the results derived from individual light metrics: Participants in Switzerland experience longer and brighter days and darker and earlier nights. While the longer photoperiod in Switzerland might partly explain the daytime effects, it does not explain the described nighttime effects.

Despite this strong individual variation, we can still reject H0_1_ and H0_2_ of our first research question since we did find statistical differences in light-logger-derived light exposure intensity levels and duration of intensity, as well as timing of light exposure, between Malaysia and Switzerland (see also Table S10).

Furthermore, there is strong evidence for a site-specific effect of sex. While there is no difference in overall melanopic EDI due to sex, the patterns of exposure differ. Specifically, females in Malaysia are less exposed to light in the hours before noon and around midnight, compared to males. In Switzerland, females are exposed to more light before noon, compared to males, and are also more likely to have a higher exposure in the afternoon. The effect size is a reduction of about 60% for women in Malaysia in the specified times of day, and about a 5-fold increase for women in Switzerland before noon, and about 2- to 3-fold in the afternoon. See Fig. S2 for further details on sex-dependent patterns and differences.

Because of the significant age difference between sites, age is a confounder for comparison between the sites (see Fig. S3). Thus, we did not explore how age relates to light exposure in general, but rather whether age differences from the site-mean affect light exposure. These results will have to be taken very tentatively, as the data is sparse. Even when corrected for the age difference, the overall difference pattern between the sites remained: Participants in Switzerland exhibit brighter days, particularly in the morning, and darker nights. There is some evidence that younger participants (5 years below average) in Malaysia have brighter mornings and darker nights, whereas younger participants in Switzerland have brighter times around and after noon, as well as darker evenings, each compared to their average age group. Results for older participants (20 years above site average) are not reported here, as there are only one and three participants in that age group in Malaysia and Switzerland, respectively, prohibiting generalisations. Results are still shown in the full analysis supplement (https://tscnlab.github.io/BillerEtAl_JExpoSciEnvironEpidemiol_2025/).

Finally, light exposure patterns differ due to photoperiod, which could only be analysed in the Swiss dataset (see Fig. 1). Unsurprisingly, light exposure is higher and ends later in the day when photoperiods are longer. However, while the shift in exposure end is somewhat pronounced, it is very moderate in the morning. Across all photoperiod durations, there are two high points during the day, one around noon and the second in the afternoon around 4 p.m., with dips in between (see Fig. S4).

### LEBA responses do not differ between Malaysia and Switzerland and are stable over time

To address the second research question, we compared LEBA items and scores across sites and found that under the strict p-value adjustment for H3 (*n* = 28), none of the five LEBA factors or individual items were significantly different between sites (for all: *p* > 0.3; see section 5.3.1 in the full analysis document linked in Supplementary material). Thus, we cannot reject our H0_3_ for this research question since we could not find strong differences in Malaysian and Swiss LEBA scores.

Looking at the stability of LEBA scores across time within the Malaysia site (H4), bootstrapping analyses showed that scores were very stable over 1 month. All 23 questions and 1 out of 5 factors did not vary significantly in more than 50% of participants (Table S11). We consequently cannot reject H0_4_ and assume scores to be stable over time, at least in this dataset (see Table ‘Average change in LEBA metrics across 4 weeks of data collection’ in section 5.3.2. of the full analysis document linked in Supplementary material).

### LEBA items correlate with preselected light-logger-derived light exposure variables in Switzerland but not in Malaysia

**Fig. 5:**
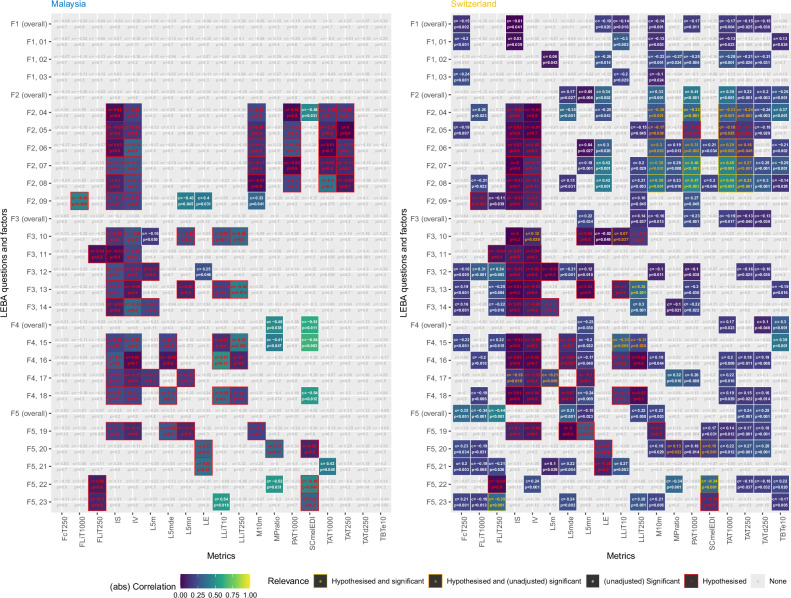
Correlations between individual LEBA items and pre-selected light metrics for Malaysia and Switzerland. Abbreviations: FcT250 Frequency crossing threshold at 250 lux, FLiT1000 Frequency of light exposure above 1000 lux, FLiT250 Frequency of light exposure above 250 lux, IS Interdaily Stability, IV Intradaily Variability, L5m Mean light exposure during the least active 5 h, L5mde Mean duration of exposure during the least active 5 h, L5mn Number of exposures during the least active 5 h, LE Light Exposure, LEBA Light Exposure Behaviour Assessment, LLiT10 Lowest light exposure intensity threshold at 10 lux, M10m Mean light exposure during the most active 10 h, MPratio Ratio of mean photopic to melanopic light exposure, PAT1000 Period above threshold at 1000 lux, SCmelEDI Spectral composition of melanopic equivalent daylight illuminance, TAT1000 Time above threshold at 1000 lux, TAT250 Time above threshold at 250 lux, TATd250 Time above dynamic threshold at 250 lux, TBTe10 Time below threshold at 10 lux.

While exploratory analysis shows correlations in both sites, only Switzerland shows significant correlations after correction for multiple testing (Fig. 5). Out of 84 correlations hypothesised a priori, 10 were significant in Switzerland.

The effect size of those correlations was medium on average (*r* = 0.39), and all correlations showed the hypothesised direction (Table 2). We can thus reject H0_5_ since we did indeed observe statistically significant correlations between LEBA and light-logger-derived variables.

**Table 2:** Significant correlations between individual LEBA items and hypothesised light metrics for Malaysia and Switzerland.

Question/Factor	Metric	LEBA item	Correlation coefficient^a^	*p*-value^b^	Interpretation
I spend 30 min or less per day (in total) outside	PAT1000	F2, 04	−0.33	0.018	The more often 30 min or less are spent outside, the shorter the period above 1000 lx
I spend more than 3 h per day (in total) outside	PAT1000	F2, 07	0.46	<0.001	The more often >3 h per day are spent outside, the longer the period above 1000 lx
I spend more than 3 h per day (in total) outside	TAT1000	F2, 07	0.45	<0.001	The more often > 3 h per day are spent outside, the higher the time above 1000 lx
I spend as much time outside as possible	M10m	F2, 08	0.39	0.003	The more spend time outside as possible, the brighter the brightest 10 h (mean)
I spend as much time outside as possible	PAT1000	F2, 08	0.47	<0.001	The more spend time outside as possible, the longer the period above 1000 lx
I spend as much time outside as possible	TAT1000	F2, 08	0.48	<0.001	The more spend time outside as possible, the higher the time above 1000 lx
I spend as much time outside as possible	TAT250	F2, 08	0.32	0.011	The more spend time outside as possible, the higher the time above 250 lx
I look at my smartwatch within 1 h before attempting to fall asleep	LLiT250	F3, 13	0.34	<0.001	The more often smartwatches are looked at within 1h before attempting to fall asleep, the later the last time above 250 lx
I use an alarm with a dawn simulation light	SCmelEDI	F5, 22	−0.34	<0.001	The more an alarm with a dawn simulation light is used, the warmer the light colour temperature
I turn on the lights immediately after waking up	FLiT250	F5, 23	−0.35	0.002	The more often the lights are turned on immediately after waking up, the earlier the first time above 250 lx

*FLiT*250 first time above threshold of 250 lux, *LLiT*250 last time above threshold of 250 lux, *M*10*m* mean light exposure during the most active 10 h, *PAT*1000 period above threshold at 1000 lux, *SCmelEDI* spectral composition of equivalent daylight illuminance, *TAT*1000 time above threshold at 1000 lux, *TAT*250 time above threshold at 250 lux.

*P*-values are considered significant below 0.05.

^a^Spearman correlation coefficient between the metric and the LEBA question/factor.

^b^*p*-values are adjusted for multiple comparisons using the false-discovery-rate for *n *= 168 comparisons.

There were also a few LEBA questions where the correlation coefficients differed significantly between sites. Specifically, these were the questions ‘*I dim my mobile phone screen within 1 hour before attempting to fall asleep’* (F4 item 15) and ‘*I dim my computer screen within 1 hour before attempting to fall asleep’* (F4 item 18; Fig. 5). While the correlation is positive with preselected light exposure metrics in Malaysia (*r* = 0.25 both, *p* = 0.019), it is zero or negative in Switzerland (*r* = –0.01 and –0.15, respectively, *p* = 0.035; Table S12). We can, therefore, also reject H0_6_, given that we observed differences in the strength and directions of LEBA scores/items and light-logger-derived variable correlations between Malaysia and Switzerland.

## Discussion

In this observational study, we investigated light exposure and light exposure behaviour differences in 39 participants in their everyday lives over 30 consecutive days. Participants were recruited in Basel, Switzerland, and Kuala Lumpur, Malaysia, two locations that differ in culture, climate, urban environment and photoperiod. Given the multitude of available light metrics to quantify objective light exposure and a lack of standards on which of those metrics are to be reported [45, 46], we also investigated which specific light metrics differed between these sites (**RQ 1**).

### Objective light exposure is different between Switzerland and Malaysia beyond the differences in photoperiod

We found that participants in Switzerland spent significantly more time in bright light levels likely corresponding to daylight (i.e. >1000 lux melanopic EDI) than participants in Malaysia during the day. Participants in Switzerland also stayed longer and about twice as often in recommended light levels during the day (i.e. >250 lux melanopic EDI). This is generally expected, given the longer photoperiod in Switzerland compared to the Malaysian site (see Fig. 1D). However, these findings still hold when adjusting for the longer photoperiod in Switzerland, indicating other differences between the sites. Furthermore, the brightest time of day was significantly brighter for participants in Switzerland compared to participants in Malaysia, with a difference of about 0.5 log_10_ units, indicating that participants in Switzerland experienced their brightest time of day with an intensity approximately 3.16 times higher. This potentially hints towards differences in behaviour since theoretical access to daylight based on photoperiod was comparable, although shorter photoperiods also offer lower daylight levels. A more likely explanation might be, though, that in both sites, melanopic EDI levels during the day indicate predominantly indoor environments. Still, the significantly higher and longer exposure to bright light in participants in Switzerland suggests better access to daylight while being indoors, compared to Malaysia.

As expected, the last time of day with bright light exposure above 250 lux melanopic EDI was later by about 1.5 h in Switzerland compared to Malaysia, which can partially be explained by the later dusk in Switzerland (about 0.8 h), but also by daylight savings time (DST), which was in place in Switzerland during data collection, but not in Malaysia. This means that participants in Switzerland experienced light until later in their day, which could contribute to later chronotypes (although the reverse could also be true). Previous studies have shown an interaction of chronotype and light exposure as well as sleep timings and light exposure [47–51]. Unfortunately, chronotype was not formally assessed in this study, but based on PSQI-derived mid-sleep (MS) as a proxy for chronotype, we also found a correlation between chronotype and mean timing of light above 250 mel EDI in Malaysia (*r* = 0.22, *p* < 0.001) but not in Switzerland (*r* = 0.05, *p* = 0.27). Note that we did not categorise chronotype into ‘early’, ‘late’, or ‘intermediate’ types [52] since this type of categorisation is not particularly meaningful in our case, spanning two very distinct geographic locations and cultures. According to this binning, mid-sleep before 3 a.m. is ‘early’, while mid-sleep after 4 a.m. is ‘late’ (while both early and late have sub-categories such as ‘moderate’ or ‘definite’). Applying these categories across sites would reduce the difference to a significantly higher proportion of early types in Switzerland compared to Malaysia. This ignores several important distinctions that are retained in the continuous scale: the distribution of chronotypes between sites (Fig. S5, and Fig. 2) shows a clear difference in the median but also scale. Except for one particularly late participant in Switzerland, Swiss participants do not exhibit a large variance compared to Malaysia. The phase of the photoperiod is different between the sites (e.g., based on solar noon), explaining about one-fifth of the difference in the mean. Thus, other factors must contribute to this difference and the wider distribution as well. For example, a strong daytime stimulus of natural daylight and low levels of artificial light at night have been shown to both reduce the variance of melatonin rhythms (which are also used as a proxy for chronotype), and result in a shift towards earlier rhythms, i.e. earlier chronotypes [49, 53].

Participants in Switzerland in our study also avoided light exposure above 10 lux mel EDI significantly earlier than participants in Malaysia by about 1h and 10 min (i.e., the timing of brighter light exposure in the evening). This means that although they still experienced some light later in their 24 day, it was dimmer than the light participants in Malaysia experienced before their bedtime. Additionally, participants in Switzerland averaged about five times (~0.6 log_10_ units) lower melanopic EDI levels during evenings, suggesting that participants in Switzerland seem to have actively avoided bright light exposure or preferred dimly lit environments. The latter could be explained by cultural differences in light preference, with a potential preference for brighter and whiter electric light in Asian cultures [54]. We cannot provide evidence beyond speculation here, as we did not assess participants’ lighting preferences.

### Objective light exposure patterns reveal strong differences between individuals within the sites, but do not follow recommendations for healthy light exposure in either site

Our analysis of light exposure patterns reveals that while, on average, the above-mentioned differences in light exposure hold up, the explanatory power of a single exposure pattern per site is negligible. Instead, each site consists of several individual patterns, with a within-site variation greater than between sites. This is encouraging, as it means people on an individual level have influence over their light exposure pattern beyond their environmental or cultural baseline. Thus, interventions to modify personal light exposure patterns might have a greater chance of success than they would have with very similar individual patterns across each site. While we did not assess socioeconomic status (SES), it should be noted that Malaysia and Switzerland show different ranges in GDP per capita, differing by almost a factor of 10 in 2024 (Malaysia: $11,867.3 vs. Switzerland: $103,669.9) [55]. This is important because, although there is no systematic data on how SES might affect light exposure, there are indications that light exposure at night depends on SES [56, 57].

While light exposure was more favourable in the Swiss dataset, neither participants in Switzerland nor Malaysia spent much of their time in bright light above 250 lux melanopic EDI, as recommended by Brown and colleagues [16]. For participants in Switzerland, it is about 12 min per hour of daylight, and 7 min for participants in Malaysia, i.e. 20% and 12% of the available daytime, respectively, which corroborates previous findings where participants in Switzerland spent only 14% of their light logger wearing time above 250 lux melanopic EDI during daytime [25]. In the evening, the participants in Switzerland in our study reduced their light exposure level earlier to the recommended 10 lux melanopic EDI or below. Still, both sites had times of elevated light exposure levels after dusk.

### Self-reported light exposure behaviour does not differ between Switzerland and Malaysia and is stable over time

A second goal of this study was to understand if subjectively reported light exposure behaviour measured with the LEBA instrument showed cultural differences between the two locations (**RQ 2**). Since LEBA is a new instrument, cultural differences, also in specific items or scores of the questionnaire, are yet to be investigated and tested. In contrast to our expectations, LEBA items and factors did not show a significant difference between Malaysia and Switzerland, meaning that light exposure behaviour assessed with the LEBA was comparable across these cultures, at least in our sample. Due to the timeframe overlap in the Malaysian dataset (i.e. participants were asked every few days about the previous 4 weeks), we were also able to investigate if items and scores were stable over time, which we confirmed for most of the LEBA items. All 23 questions and 1 out of 5 factors did not vary significantly in more than 50% of participants. While the other factors showed variation over time, it was very small, with an average sum of 3-to-4-point total differences across nine survey periods. Unfortunately, we were unable to do this analysis in the Swiss dataset because the timespans used for the instruction in the LEBA questionnaires were not comparable. Therefore, we cannot be sure if the stability of LEBA items can be generalised beyond the Malaysian site, but we would hypothesise so based on the overall lack of differences between cultures in LEBA items and scores.

### Self-reported light exposure behaviour is reflected in objective light exposure but only in participants in Switzerland

Finally, we wanted to test if subjectively reported light exposure behaviour can be related to objectively measured light exposure and, again, if these relationships might depend on location (**RQ3**). The results clearly show that there are strong correlations between certain light exposure metrics and answers to the LEBA questionnaire, but only in the Swiss dataset. After adjustment for multiple comparisons, none of the correlations in the Malaysian dataset remained significant. Interestingly, the average (absolute) correlation was stronger in the Malaysia site than in Switzerland, indicating a higher variance in either light exposure metrics or LEBA answers in the Malaysia dataset compared to Switzerland. For the Swiss dataset, ten of the hypothesised correlations were shown to be significant after adjustment with an average medium effect size [58, 59].

We acknowledge that differences in the sites might also be due to the different phrasing of the LEBA: whereas it questioned participants about the *past few days* in Switzerland, it asked about the *past four weeks* in Malaysia. Thus, the differences in correlative strength might also be due to participants being able to recollect light exposure-related behaviour better for a few days compared to the last month.

### Strength and weaknesses

Our study has several strengths and weaknesses. We sampled data from a relatively small cohort of *n* = 19 and *n* = 20 in Malaysia and Switzerland, respectively, without controlling for SES, body mass index (BMI) or mental/psychiatric disorders, which were previously shown to alter light exposure [56, 57, 60–63]. However, given the longitudinal data of 4 weeks per participant, we are confident that we have enough data points to rely on the model results that we presented. Since we only collected data from one site per country, we cannot be sure that these sites represent the entire country, but this was also not the overarching research goal. Unfortunately, we lacked information on chronotype, weekday type information (work day vs. free day), as well as type of activity or work schedules, so we could not compute statistics that would have included such information, despite previous studies showing their potential influence [64]. The light logger we used, the SPECCY wearable, also did not record activity (actimetry data) to save battery, so we could not use this information to detect the non-wear time of the device, nor estimate chronotype based on activity. We have, however, approximated chronotype based on mid-sleep from the PSQI questionnaire.

Our study, adds to the novel literature on light exposure *behaviour* and is the first to relate subjectively assessed light behaviour to objectively recorded light exposure behaviour. Our data sampling is both longitudinal and takes place at high frequency, allowing for fine-grained and highly reliable data analysis. By positioning the light logger at chest level, we were able to better approximate physiologically relevant light exposure than at wrist positions. Finally, we add to the growing literature on individual and cultural differences, which need to be factored in for better and fine-tuned recommendations and provide here a dataset that generated novel hypotheses that can be tested explicitly in the future.

## Conclusion

This study offers a detailed investigation into the differences in light exposure and light exposure behaviour between two distinct geographical and cultural contexts: Switzerland and Malaysia. Key findings reveal significant differences in objective light exposure between the two sites. Participants in Switzerland experienced brighter and longer daylight exposure, even when accounting for photoperiod differences, while avoiding evening light exposure earlier and to a greater extent than participants in Switzerland. These results suggest behavioural and cultural preferences or differences in access to daylight, all of which influence individual light exposure patterns. Additionally, our finding that subjective light exposure behaviour correlates with objective light metrics only in the Swiss dataset underscores potential cultural or methodological differences in self-reporting accuracy. Despite this, the LEBA instrument proved stable over time, offering preliminary evidence for its reliability.

While our study advances the understanding of light exposure behaviours and cultural nuances, it also highlights several areas for future exploration. Investigating preferences for electrical light colour and intensity could also clarify the cultural differences hypothesised in this study. Expanding the sample size and geographic scope would improve the generalisability of these findings, while further validation of the LEBA instrument in diverse populations is essential to confirm its cross-cultural applicability and whether it could be used as an approximation for objectively assessed light exposure. Similarly, we recommend that future studies include SES as a confounding variable and even systematically study differences in light exposure based on SES [65].

From an applied perspective, these findings have implications for designing culturally informed light exposure guidelines to promote healthier circadian alignment. The apparent avoidance of evening light exposure in the participants in Switzerland aligns with recommendations for better sleep and circadian health. In contrast, the ‘preferences’, or at least exposure patterns, observed among the participants in Malaysia, highlight the need for interventions aimed at reducing evening light exposure. These could include promoting the use of warmer, dimmer lighting in the evening and adjusting the timing of brighter light exposure during the day. Such strategies should consider local preferences and location-specific constraints, such as Malaysia’s daytime heat, which often discourages outdoor activities and exposure during daylight hours. Future studies should explore how environmental and behavioural interventions could optimise light exposure for health outcomes across diverse populations and climates to be feasible in the real world beyond Western locations and in light of global warming.

In conclusion, this study provides a foundation for understanding the interaction of light exposure behaviour, culture, and geography, opening new avenues for research and intervention strategies. By addressing the identified limitations and building on the strengths of our approach, future research can continue to uncover actionable insights into the critical role of light exposure in human circadian and sleep health.

## Supplementary information

Supplementary information

## Data Availability

Data and code are available on https://github.com/tscnlab/BillerEtAl_JExpoSciEnvironEpidemiol_2025 and are archived on Zenodo (10.5281/zenodo. 16872096) under a permissive CC-BY 4.0 Attribution license. The webpage https://tscnlab.github.io/BillerEtAl_JExpoSciEnvironEpidemiol_2025/ contains a Notebook of code and code outputs.
