# Supplementary material for: Physiologically-relevant light exposure and light behaviour in Switzerland and Malaysia
Source: J Expo Sci Environ Epidemiol. 2025 Dec 3;36(2):409–22. doi: 10.1038/s41370-025-00825-8 (PMC12960204; doi:10.1038/s41370-025-00825-8)
Supplement: Supplementary file 1 — Supplementary information [file 41370_2025_825_MOESM1_ESM.pdf]

Physiologically-relevant light exposure and light behaviour in Switzerland and Malaysia

Supplementary Material

Authors

Anna M Biller*[1,2] [0000-0002-3673-8838]

Johannes Zauner*[1,2] [0000-0003-2171-4566]

Christian Cajochen[3] [0000-0003-2699-7171]

Marisa A Gerle[3] [0009-0000-9159-1093]

Vineetha Kalavally[4] [0000-0002-3531-9076]

Anas Mohamed[4] [0000-0001-5418-4501]

Lukas Rottländer[3] [0009-0007-9062-0113]

Ming-Yi Seah[4] [0009-0005-5409-5839]

Oliver Stefani[5] [0000-0003-3854-3222]

Manuel Spitschan[1,2,6] [0000-0002-8572-9268]

Affiliations

1 Technical University of Munich, TUM School of Medicine and Health, Department Health and Sport Sciences, Chronobiology & Health, Munich, Germany

2 Translational Sensory & Circadian Neuroscience, Max Planck Institute for Biological Cybernetics, Tübingen, Germany

3 Centre for Chronobiology, University Psychiatric Clinics Basel (UPK), Basel, Switzerland

4 Monash University Malaysia, Electrical and Computer Systems Engineering, Intelligent Lighting Laboratory, Malaysia

5 Lucerne School of Engineering and Architecture, Lucerne University of Applied Sciences and Arts, Horw, Switzerland

6 TUM Institute for Advanced Study (TUM-IAS), Technical University of Munich, Garching, Germany

Supplementary material

Supplementary Tables

Factor name	Score
F1: Wearing blue light filters	01+02+03
F2: Spending time outdoors	04(R)+05+06+07+08+09
F3: Using phone and smartwatch in bed	10+11+12+13+14
F4: Using light before bedtime	15+16+17+18
F5: Using light in the morning and during daytime	19+20+21+22+23

Table S1. Scoring of Light Exposure Behaviour Assessment (LEBA) items. Note that R denotes items that are reversed-scored. F1-F5 denotes the calculated Factors.

Component	Question (Q)	Component Score
**(1)** Subjective sleep quality	#6	0-3
**(2)** Sleep latency	#2<br>#5a	Sum of Q2 (0-3)+Q5a (0-3):<br>If 0 → 0<br>1-2 → 1<br>3-4 → 2<br>5-6 → 3
**(3)** Sleep duration	#4	0-3
**(4)** Habitual sleep efficiency<br>(Sleep efficiency = hours slept / hours in bed) X 100%)	#1<br>#3<br>#4	If >85% → 0<br>75-84% → 1<br>65-74% → 2<br><65% → 3
**(5)** Sleep disturbance	#5b-5j	Sum of Q5b (0-3) to Q5 (0-3):<br>If 0 → 0<br>1-9 → 1<br>10-18 → 2<br>19-27 → 3
**(6)** Use of sleep medication	#7	0-3
**(7)** Daytime dysfunction	#8<br>#9	Sum of Q8 (0-3) +Q9 (0-3:<br>If 0 → 0<br>1-2 → 1<br>3-4 → 2<br>5-6 → 3
**Global PSQI Score**		**Sum of Components 1-7**

Table S2. Scoring of the Pittsburgh Sleep Quality Index (PSQI) items.

Call to calculate metric	Light metric name	Description
bright_dark_period()	Brightest or darkest continuous period	Finds the brightest or darkest continuous period of a given timespan and calculates its mean light level, as well as the timing of the period's onset, midpoint, and offset. It is defined as the period with the maximum or minimum mean light level. Note that the data need to be regularly spaced (i.e., no gaps) for correct results
duration_above_threshold()	Duration above/below threshold or within threshold range	Calculates the duration spent above/below a specified threshold light level or within a specified range of light levels
frequency_crossing_threshold()	Frequency of crossing light threshold	Calculates the number of times a given threshold light level is crossed
intradaily_variability()	Intradaily variability (IV)	Calculates the variability of consecutive light levels within a 24h day. Calculated as the ratio of the variance of the differences between consecutive light levels to the total variance across the day. Calculated with mean hourly light levels. Higher values indicate more fragmentation
interdaily_stability()	Interdaily stability (IS)	This function calculates the variability of 24h light exposure patterns across multiple days. Calculated as the ratio of the variance of the average daily pattern to the total variance across all days. Calculated with mean hourly light levels. Ranges between 0 (Gaussian noise) and 1 (Perfect Stability)
period_above_threshold()	Length of longest continuous period above/below threshold	Length of the longest continous period above/below a specified threshold light level or within a specified range of light

		levels
pulses_above_threshold()	Pulses above threshold	Clusters the light data into continuous clusters (pulses) of light above/below a given threshold
threshold_for_duration()	Find threshold for given duration	Threshold for which light levels are above/below for a given duration. This function can be considered as the inverse of duration_above_threshold
timing_above_threshold()	Mean/first/last timing above/below threshold.	Mean, first, and last timepoint (MLiT, FLiT, LLiT) where light levels are above or below a given threshold intensity within the given time interval

Table S3. Overview of objectively derived outcome variables from light logging data and their calculations.

RQ #	Hypothesis	Variable(s)	Statistical test(s)	Wilkinson Formula (if appropriate)
**1**	**H1:** There are differences in light logger-derived light exposure **intensity levels** and **duration** of intensity between Malaysia and Switzerland.<br><br>**H0$_1$:** No difference.	Intensity level light metrics<br>- TAT250<br>- TAT1000<br>- Period above threshold 1000<br>- TAT250 (daytime hours)<br>- TBT10 (evening hours)	Generalized mixed-effect analyses on the effect of site on various light parameters<br><br>5 Models	metric = site + (1|site:participant)
	**H2:** There are differences in light logger-derived **timing of light** exposure between Malaysia and Switzerland.<br><br>**H0$_2$:** No difference.	Timing light metrics<br>- M10m<br>- L5m<br>- IS<br>- IV<br>- LLiT 10<br>- LLit 250<br>- Frequency crossing threshold	Generalized mixed-effect model (GAMM) on the effect of site on various parameters<br><br>7 models	metric = site + (1|site:participant)
		Time-of-day (two-level factor: daytime / evening)<br>Melanopic EDI	Linear mixed-effect model on the interaction of time-of-day and site on melanopic EDI	melEDI = site · time-of-day + (1 + time-of-day | site:participant)
		Time-of-day (seco from midnight)<br>Melanopic EDI	Generalized mixed-effect model (GAMM) on the interaction of time-of-day and site on melanopic EDI	melEDI = s(time-of-day, site) + s(time-of-day, by = participant) + s(participant, by = site)<br><br>Note on GAMM basis splines for smooth (*s()*) terms:<br>1. cyclic spline (cs), factor spline (fs)<br>2. cyclic spline (cs)<br>3. random effect (re)
**2**	**H3:** There are differences in LEBA items and factors between Malaysia and Switzerland.	- all individual LEBA items (**Table 5**)<br>- all 5 LEBA Factors (**Table 2**)	Cumulative link mixed-effect analyses on the effect of site on each LEBA question and factor	LEBA item/factor = site + (1 | site:participant)

	**$H0_3$:** No difference.		23 + 5 models	
	**H4:** LEBA scores vary over time within participants<br><br>**$H0_4$:** no variance	- all individual LEBA items (**Table 5**)<br>- all 5 LEBA Factors (**Table 2**)	Bootstrap analysis of the standard deviation of LEBA scores within participants for the Malaysia dataset<br><br>23 + 5 bootstraps	
**3**	**H5:** LEBA items correlate with pre-selected light logger-derived light exposure variables.<br><br>**$H0_5$:** No correlation.	See **Table 5** for all relevant variables.	Correlation matrix with one matrix per site.<br><br>2x85 correlations	
	**H6:** There is a difference between Malaysia and Switzerland on how well light logger-derived light exposure variables correlate with subjective LEBA items.<br><br>**$H0_6$:** No difference between Malaysia and Switzerland.	See **Table 5** for all relevant variables.	Generalized mixed-effect analyses to determine the effect of site on the dependency between light exposure metrics and LEBA items/factors<br><br>23 + 5 models	$\text{metric} = \text{site} \cdot \text{LEBA item/factor} + (1 \mid \text{site:participant})$

Table S4. Overview of research questions, hypotheses and statistical tests to address the hypotheses. Abbreviations: GAMM, Generalised additive mixed-effect model; $H0_x$, Null hypothesis; HX, Hypothesis X; IS, Interdaily Stability; IV, Intradaily variability; LEBA, Light Exposure Behaviour Assessment; LLiT, Last time above threshold; LLiT 10, Low light intensity threshold at 10 lux; LLiT 250, Low light intensity threshold at 250 lux; L5m, Mean across darkest 5 hours; M10m, Mean across brightest 10 hours; melEDI, Melanopic equivalent daylight illuminance; RQ, Research question; TAT250, Time above threshold 250 lux; TAT1000, Time above threshold 1000 lux; TBT10, Time below threshold 10 lux.

LEBA Item	LEBA Question	List of calculated light variables
1	I wear blue-filtering, orange-tinted, and/or red-tinted glasses indoors during the day.	NA
2	I wear blue-filtering, orange-tinted, and/or red-tinted glasses outdoors during the day.	NA
3	I wear blue-filtering, orange-tinted, and/or red-tinted glasses 1 hour before attempting to fall asleep.	NA
4	I spend 30 minutes or less per day (in total) outside.	['M10', 'TAT250', 'TAT1000', 'Period above threshold 1000', 'IV', 'IS']
5	I spend between 30 minutes and 1 hour per day (in total) outside.	['M10', 'TAT250', 'TAT1000', 'Period above threshold 1000', 'IV', 'IS']
6	I spend between 1 and 3 hours per day (in total) outside.	['M10', 'TAT250', 'TAT1000', 'Period above threshold 1000', 'IV', 'IS']
7	I spend more than 3 hours per day (in total) outside.	['M10', 'TAT250', 'TAT1000', 'Period above threshold 1000', 'IV', 'IS']
8	I spend as much time outside as possible.	['M10', 'TAT250', 'TAT1000', 'Period above threshold 1000', 'IV', 'IS']
9	I go for a walk or exercise outside within 2 hours after waking up.	['first time above threshold of 1000 (FLitT 1000)', 'IV', 'IS']
10	I use my mobile phone within 1 hour before attempting to fall asleep.	['last time above threshold of 10 (LLiT 10)', 'last time above threshold of 250 (LLiT 250)', 'L5 (without sleep period)', 'IV', 'IS']
13	I look at my smartwatch within 1 hour before attempting to fall asleep.	['last time above threshold of 10 (LLiT 10)', 'last time above threshold of 250 (LLiT 250)', 'L5 (without sleep period)', 'IV', 'IS']
11	I look at my mobile phone screen immediately after waking up.	['first time above threshold of 250 (FLitT 250)', 'IV', 'IS']
12	I check my phone when I wake up at night.	['L5', 'IV', 'IS']
14	I look at my smartwatch when I wake up at night.	['L5', 'IV', 'IS']
15	I dim my mobile phone screen within 1 hour before attempting to fall asleep.	['last time above threshold of 10 (LLiT 10)', 'last time above threshold of 250 (LLiT 250)', 'L5 (without sleep period)', 'IV', 'IS']
16	I use a blue-filter app on my computer screen within 1 hour before attempting to fall asleep.	['last time above threshold of 10 (LLiT 10)', 'last time above threshold of 250 (LLiT 250)', 'L5 (without sleep period)', 'IV', 'IS']
17	I use as little light as possible when I get up during the night.	['L5 and L5 (only night)', 'IV', 'IS']
18	I dim my computer screen within 1 hour before attempting to fall asleep.	['last time above threshold of 10 (LLiT 10)', 'last time above threshold of 250 (LLiT 250)', 'L5 (without sleep period)', 'IV', 'IS']
19	I use tunable lights to create a healthy light environment.	['IV', 'IS', 'L5 (with/without night)', 'M10']
20	I use LEDs to create a healthy light environment.	['LE', 'Spectral contribution', 'Melanopic/photopic ratio']
21	I use a desk lamp when I do focused work.	['LE']
22	I use an alarm with a dawn simulation.	['Spectral contribution', 'first time above threshold of 10 (FLitT 10)', 'first time above threshold of 250 (FLitT 250)']
23	I turn on the lights immediately after waking up.	['Spectral contribution', 'first time above threshold of 10 (FLitT 10)', 'first time above threshold of 250 (FLitT 250)']

Table S5. List of Light Exposure Behaviour Assessment (LEBA) items and corresponding objectively derived light variables from light logging data. In yellow are the hypothesised and significant relationships in the Swiss dataset. Abbreviations: FLiT 10, First time above threshold of 10 lux; FLiT 250, First time above threshold of 250 lux; FLiT 1000, First time above threshold of 1000 lux; IS, Interdaily Stability; IV, Intradaily Variability; LE, Light Exposure; L5, Mean across darkest 5 hours; L5 (only night), Mean across darkest 5 hours during the night; LLiT 10, Last time above threshold of 10 lux; LLiT 250, Last time above threshold of 250 lux; M10, Mean across brightest 10 hours; TAT250: Time above threshold of 250 lux; TAT1000: Time above threshold of 1000 lux.

A

Missing data in the Malaysia dataset overall

	min	max	median	mean	total
Number of gaps	0	7	0	1	22
Duration missed by available data points	0 days	14 days	0 days	1 day	29 days
Duration covered by available data points	11 days	30 days	30 days	26 days	509 days
Number of missing data points	0	21,040	0	2,200	41,797
Number of available data points	16,604	43,996	43,211	38,627	733,907
Percentage of missing data points	0.0%	48.7%	0.0%	5.4%	5.4%

min, max, median, mean, and total values for 19 participants

B

Missing data in the Swiss dataset overall

	min	max	median	mean	total
Number of gaps	1	4	1	2	35
Duration missed by available data points	<1 day	11 days	<1 day	1 day	28 days
Duration covered by available data points	7 days	30 days	29 days	27 days	554 days
Number of missing data points	21	16,017	44	2,035	40,698
Number of available data points	11,476	44,586	42,974	39,954	799,070
Percentage of missing data points	0.0%	40.4%	0.1%	5.9%	4.8%

min, max, median, mean, and total values for 20 participants

Table S6. Overview of missing data for the two datasets. A, Shows the overview for Malaysia and B, for Switzerland. Abbreviations: Min, minimum; Max, maximum.

Model Results for Hypothesis 1					
			Site coefficients		
	p-value[1]	Intercept[2]	Malaysia[3]	Switzerland[3]	
**TAT250**	**0.002**	418	1	1.780	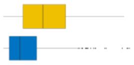
**TAT1000**	**0.011**	180	1	1.942	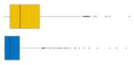
**PAT1000**	0.15	78	—	—	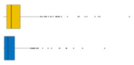
**TATd250**	**0.003**	410	1	1.772	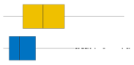
**TBTe10**	0.9	678	—	—	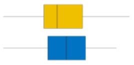

[1] p-values are adjusted for multiple comparisons using the false-discovery-rate for n= 5 comparisons
[2] Model prediction for the intercept per hour of photo- or nighttime period, reference level for the site is Malaysia
[3] Exponentiated beta coefficients from the final model, denoting the multiplication factor for the intercept, conditional on the site

Table S7. Model results for H1. P-values are considered significant below 0.05. Abbreviations: PAT1000, Period above threshold 1000, TAT250, Time above threshold of 250 lux; TAT1000, Time above threshold of 1000 lux; TATd250, Time above dynamic threshold 250 lux; TBTe10, Time below threshold 10 lux.

Model Results for Hypothesis 2, Interaction								
			**Site coefficients**		**Time coeff.**		**Interaction coeff.**	
	**p-value**[1,2]	**Intercept**	**Malaysia**	**Switzerland**	**Day**	**Evening**	**Switzerland:Evening**	
**log10(daily mean mel EDI)**	**<0.001**	2.23	0	0.41	0	−0.98	−1.11	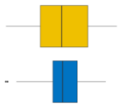

Values were log10 transformed before model fitting

[1] p-values are adjusted for multiple comparisons using the false-discovery-rate for n= 1 comparisons

[2] p-value refers to the interaction effect

Table S8. Model results for H2. P-values are considered significant below 0.05. Abbreviations: Mel EDI, melanopic equivalent daylight illumination.

Model Results for Hypothesis 2, Timing

			Site coefficients		
	p-value[1]	Intercept	Malaysia	Switzerland	
**M10m**[2]	**0.004**	2.36	0	0.46	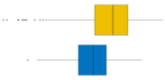
**L5m**[2]	0.2	−0.88	—	—	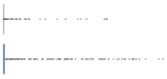
**IV**	0.9	1.28	—	—	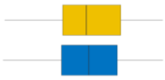
**LLiT10**	**0.005**	23:16:14	0	−01:10:42	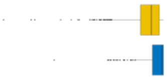
**LLiT250**	**<0.001**	17:41:23	0	01:27:54	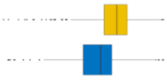
**FcT250**	**0.005**	35.79	0	28.71	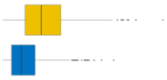
**IS**	0.9	0.16	—	—	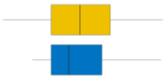

[1] p-values are adjusted for multiple comparisons using the false-discovery-rate for n= 9 comparisons

[2] Values were log10 transformed before model fitting

Table S9. Model results for H2. P-values are considered significant below 0.05. Abbreviations: FcT250, Frequency of crossing light threshold at 250 lux; FLiT 10, First time above threshold of 10 lux; FLiT 250, First time above threshold of 250 lux; FLiT 1000, First time above threshold of 1000 lux; IS, Interdaily Stability; IV, Intradaily Variability; L5m, Mean across darkest 5 hours; LE, Light Exposure; LLiT 10, Last time above threshold of 10 lux; LLiT 250, Last time above threshold of 250 lux; M10m, Mean across brightest 10 hours;

	Malaysia			Switzerland				
	Mean	SD	n	Mean	SD	n	Difference M-S	Boxplot
**FLiT1000**	**38,664**	8,857	413	**35,909**	8,818	489	**2,755**	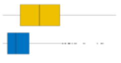
**FLiT250**	**37,192**	9,723	439	**33,204**	9,880	509	**3,988**	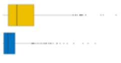
**FcT250**	**36**	31	490	**67**	44	533	**−31**	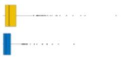
**IS**	**0.162**	0.0581	19	**0.164**	0.0664	20	**−0.00129**	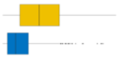
**IV**	**1.29**	0.483	472	**1.28**	0.504	504	**0.00480**	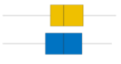
**L5m**[1]	**−0.781**	0.524	490	**−0.966**	0.183	533	**0.185**	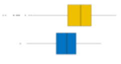
**L5mde**[1]	**0.836**	0.785	490	**0.971**	0.912	533	**−0.136**	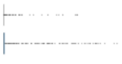
**L5mn**[1]	**−0.341**	0.897	490	**−0.797**	0.489	533	**0.456**	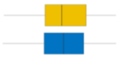
**LE**	**5,117**	7,132	490	**18,765**	26,249	533	**−13,648**	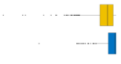
**LLiT10**	**23:18**	01:04	483	**22:18**	01:52	528	**01:00**	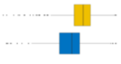
**LLiT250**	**17:42**	02:36	432	**19:14**	02:10	510	**−01:31**	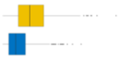
**M10m**[1]	**2.37**	0.578	490	**2.87**	0.706	533	**−0.503**	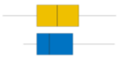
**MEDI**[1]	**0.469**	1.42	704,123	**0.547**	1.61	765,617	**−0.0788**	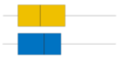
**MPratio**	**0.932**	0.119	490	**0.947**	0.103	533	**−0.0150**	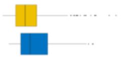
**PAT1000**	**957**	1,360	490	**1,692**	2,031	533	**−736**	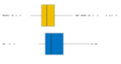
**SCmelEDI**	**1.52×10^{-3}**	1.36×10^{-4}	490	**1.40×10^{-3}**	1.19×10^{-4}	533	**1.15×10^{-4}**	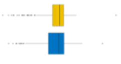
**TAT1000**	**47m**	1h 4m	490	**1h 44m**	1h 41m	533	**−57m**	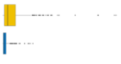
**TAT250**	**1h 38m**	1h 38m	490	**3h 28m**	2h 20m	533	**−1h 50m**	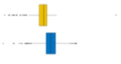
**TATd250**	**1h 36m**	1h 36m	490	**3h 25m**	2h 18m	533	**−1h 49m**	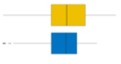
**TBTe10**	**2h 32m**	1h 5m	490	**2h 39m**	1h 1m	533	**−7m**	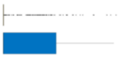

[1] values were log10 transformed

Table S10. Differences in descriptive statistics for light exposure metrics between Malaysia and Switzerland. Mean, standard deviation (SD), sample size (n) and mean differences between the two locations (M-S) are presented for each variable. Abbreviations: FcT250, Frequency of crossing light threshold at 250 lux; FLiT250, First time above threshold of 250 lux; FLiT1000, First time above threshold of 1000 lux; IS, Interdaily Stability; IV, Intradaily Variability; LE, Light Exposure; L5m, Mean across darkest 5 hours; L5mde, Mean duration of exposure during the least active 5 hours; L5mn, Number of exposures during the least active 5 hours; LLiT10, Last time above threshold of 10 lux; LLiT250, Last time above threshold of 250 lux; M10m, Mean across brightest 10 hours; mel EDI, melanopic equivalent daylight illuminance; MPratio, Ratio between melanopic and photopic illuminance; PAT1000, Period above threshold of 1000 lux; SCmelEDI, Spectal composition equivalent daylight illuminance; TAT250, Time above threshold of 250 lux; TAT1000, Time above threshold of 1000 lux; TATd250, Time above dynamic threshold of 250 lux; TBTe10, Time below threshold of 10 lux.

Hypothesis 4: Extent of stable LEBA items and factors over time
**stable over time in 75-100% of participants**
Factor 1: Item 01, Factor 1: Item 02, Factor 1: Item 03, Factor 2: Item 09, Factor 3: Item 10, Factor 3: Item 13, Factor 3: Item 14, Factor 5: Item 19, Factor 5: Item 21, Factor 5: Item 22, Factor 1
**stable over time in 50-75% of participants**
Factor 2: Item 04, Factor 2: Item 05, Factor 2: Item 06, Factor 2: Item 07, Factor 2: Item 08, Factor 3: Item 11, Factor 3: Item 12, Factor 4: Item 15, Factor 4: Item 16, Factor 4: Item 17, Factor 4: Item 18, Factor 5: Item 20, Factor 5: Item 23
**stable over time in 25-50% of participants**
Factor 3
**stable over time in 25-0% of participants**
Factor 2, Factor 4, Factor 5

Table S11. Stability of LEBA items and factors over time. For a description of each LEBA item, see Table S5.

Model Results for Hypothesis 6

	p-value†	Intercept	Site coefficients: Malaysia	Site coefficients: Switzerland
leba_F2_04	0.2	−0.07	—	—
leba_F2_05	0.9	−0.07	—	—
leba_F2_06	0.054	0.03	—	—
leba_F2_07	0.4	0.06	—	—
leba_F2_08	0.9	0.16	—	—
leba_F2_09	0.9	−0.08	—	—
leba_F3_10	0.9	0.03	—	—
leba_F3_11	0.9	0.05	—	—
leba_F3_12	0.9	0.00	—	—
leba_F3_13	0.9	0.01	—	—
leba_F3_14	0.9	−0.08	—	—
leba_F4_15	**0.019**	0.25	0	−0.40
leba_F4_16	0.9	0.09	—	—
leba_F4_17	0.9	−0.16	—	—
leba_F4_18	**0.035**	0.25	0	−0.26
leba_F5_19	0.9	−0.10	—	—
leba_F5_20	0.9	0.21	—	—
leba_F5_22	0.9	−0.20	—	—
leba_F5_23	0.9	−0.07	—	—

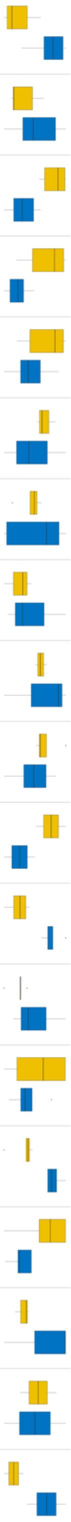

† p-values are adjusted for multiple comparisons using the false-discovery-rate for n= 28 comparisons

Table S12. Model results of H6. P-values are considered significant below 0.05. For a description of each LEBA item, see Table S5.

Characteristic	Malaysia N = 19[1]	Switzerland N = 20[1]	p-value[2]
Subjective sleep quality			>0.9
0	3 (16%)	3 (15%)	
1	15 (79%)	16 (80%)	
2	1 (5.3%)	1 (5.0%)	
3	0 (0%)	0 (0%)	
Sleep latency			>0.9
0	7 (37%)	6 (30%)	
1	9 (47%)	10 (50%)	
2	3 (16%)	3 (15%)	
3	0 (0%)	1 (5.0%)	
Sleep duration			0.2
0	4 (21%)	9 (45%)	
1	13 (68%)	11 (55%)	
2	1 (5.3%)	0 (0%)	
3	1 (5.3%)	0 (0%)	
Habitual sleep efficiency			0.020*
0	18 (95%)	12 (60%)	
1	1 (5.3%)	8 (40%)	
2	0 (0%)	0 (0%)	
3	0 (0%)	0 (0%)	
Sleep disturbance			0.5
0	2 (11%)	2 (10%)	
1	17 (89%)	16 (80%)	
2	0 (0%)	2 (10%)	
3	0 (0%)	0 (0%)	
Use of sleep medication			>0.9
0	18 (95%)	17 (85%)	
1	1 (5.3%)	2 (10%)	
2	0 (0%)	1 (5.0%)	
3	0 (0%)	0 (0%)	
Daytime dysfunction			0.4
0	3 (16%)	7 (35%)	
1	11 (58%)	9 (45%)	
2	4 (21%)	4 (20%)	
3	1 (5.3%)	0 (0%)	
Global PSQI	5.00 (2.00,9.00)	4.00 (1.00,10.00)	0.8

[1] n (%); Median (Min,Max)
[2] *p<0.05; **p<0.01; ***p<0.001

Table S13. Pittsburgh Sleep Quality Index comparisons between Malaysia and Switzerland at Day 0. P-values are considered significant below 0.05.

Characteristic	Malaysia N = 19[1]	Switzerland N = 20[1]	p-value[2]
Subjective sleep quality			0.8
0	2 (11%)	3 (17%)	
1	13 (68%)	13 (72%)	
2	4 (21%)	2 (11%)	
3	0 (0%)	0 (0%)	
missing	0	2	
Sleep latency			0.7
0	8 (42%)	5 (28%)	
1	7 (37%)	9 (50%)	
2	2 (11%)	3 (17%)	
3	2 (11%)	1 (5.6%)	
missing	0	2	
Sleep duration			0.2
0	8 (42%)	13 (72%)	
1	8 (42%)	5 (28%)	
2	2 (11%)	0 (0%)	
3	1 (5.3%)	0 (0%)	
missing	0	2	
Habitual sleep efficiency			>0.9
0	16 (84%)	17 (94%)	
1	2 (11%)	1 (5.6%)	
2	1 (5.3%)	0 (0%)	
3	0 (0%)	0 (0%)	
missing	0	2	
Sleep disturbance			0.6
0	0 (0%)	0 (0%)	
1	18 (95%)	16 (89%)	
2	1 (5.3%)	2 (11%)	
3	0 (0%)	0 (0%)	
missing	0	2	
Use of sleep medication			>0.9
0	18 (95%)	18 (100%)	
1	1 (5.3%)	0 (0%)	
2	0 (0%)	0 (0%)	
3	0 (0%)	0 (0%)	
missing	0	2	
Daytime dysfunction			0.3
0	3 (16%)	7 (39%)	
1	11 (58%)	7 (39%)	
2	4 (21%)	4 (22%)	
3	1 (5.3%)	0 (0%)	
missing	0	2	
Global PSQI	5.00 (1.00,11.00)	3.50 (2.00,8.00)	0.15
missing	0	2	

[1] n (%); Median (Min,Max)
[2] *p<0.05; **p<0.01; ***p<0.001

Table S14. Pittsburgh Sleep Quality Index comparisons between Malaysia and Switzerland on Day 31. P-values are considered significant below 0.05.

Supplementary Figures

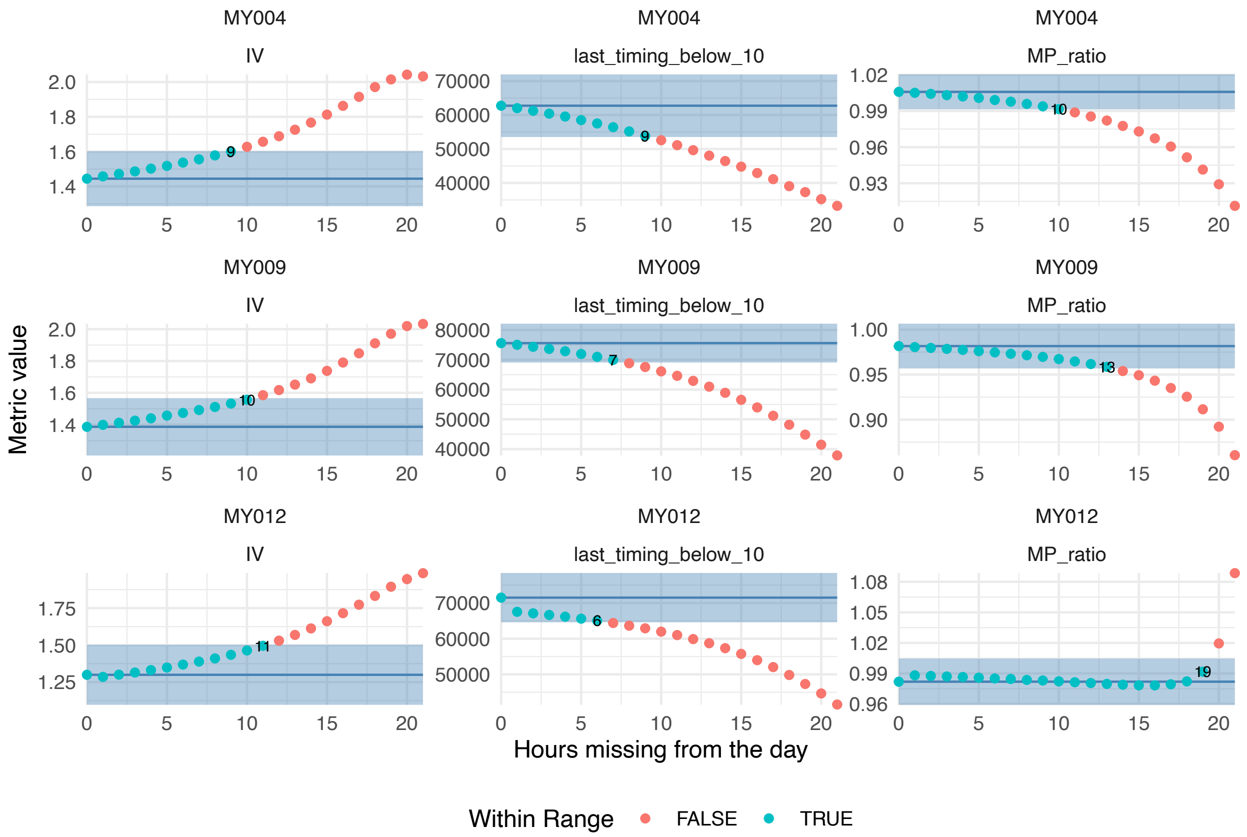

Figure S1. Summary of the bootstrapping procedure to determine an acceptable threshold of daily missing. Data based on three individual participants from the Malaysia site (MY004, MY009, MY012). Three variables were randomly chosen (IV, last time below 10 lux mel EDI, MP ratio). The blue horizontal line shows the average value of data across the full dataset, the blue rectangle a 95% confidence interval around that average. Dots show the average value across all bootstraps of that respective threshold value. A blue dot indicates that all 10^4 bootstraps lie within the 95% confidence interval of the full dataset. A red dot indicates that at least one bootstrap lies outside of the 95% confidence interval. Abbreviations: IV; Interdaily variability; MP ratio, Melanopic/photopic ratio.

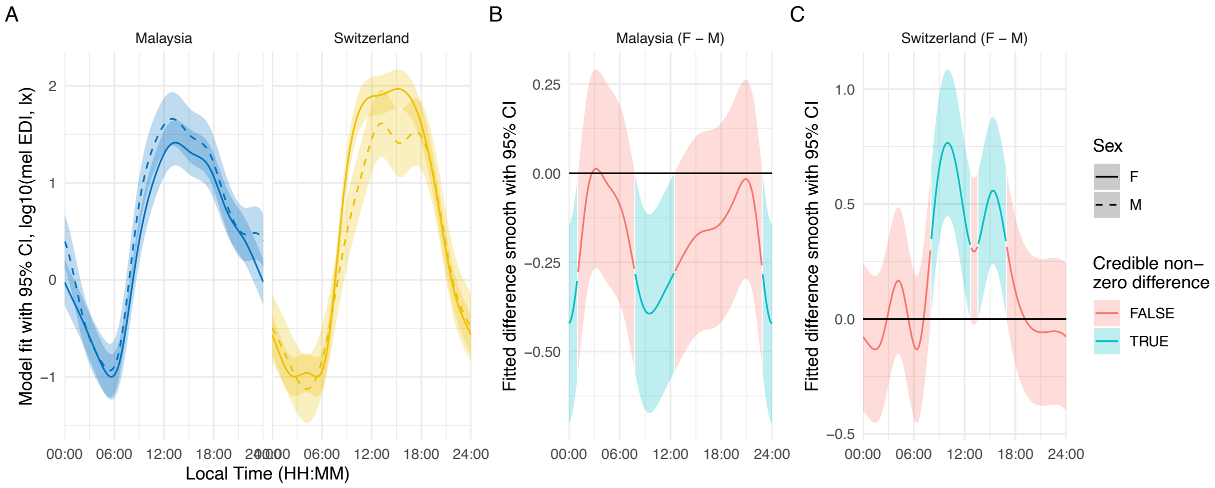

Figure S2. Sex differences in light exposure patterns. Results from a generalized additive mixed model looking at light exposure patterns across the day, dependent on site and sex. A: Model predictions for log10 melanopic EDI depending on local time, for both sites (panels) and sexes (solid and dashed lines). Bands indicate 95% confidence intervals. B: Difference smooth of log10 melanopic EDI between females and males in Malaysia. Red lines and bands indicate a non-significant differences, blue lines and bands a significant one. Values above 0 indicate higher illuminance for females at a given time, values below 0 vice versa. C: like B, but for Switzerland.

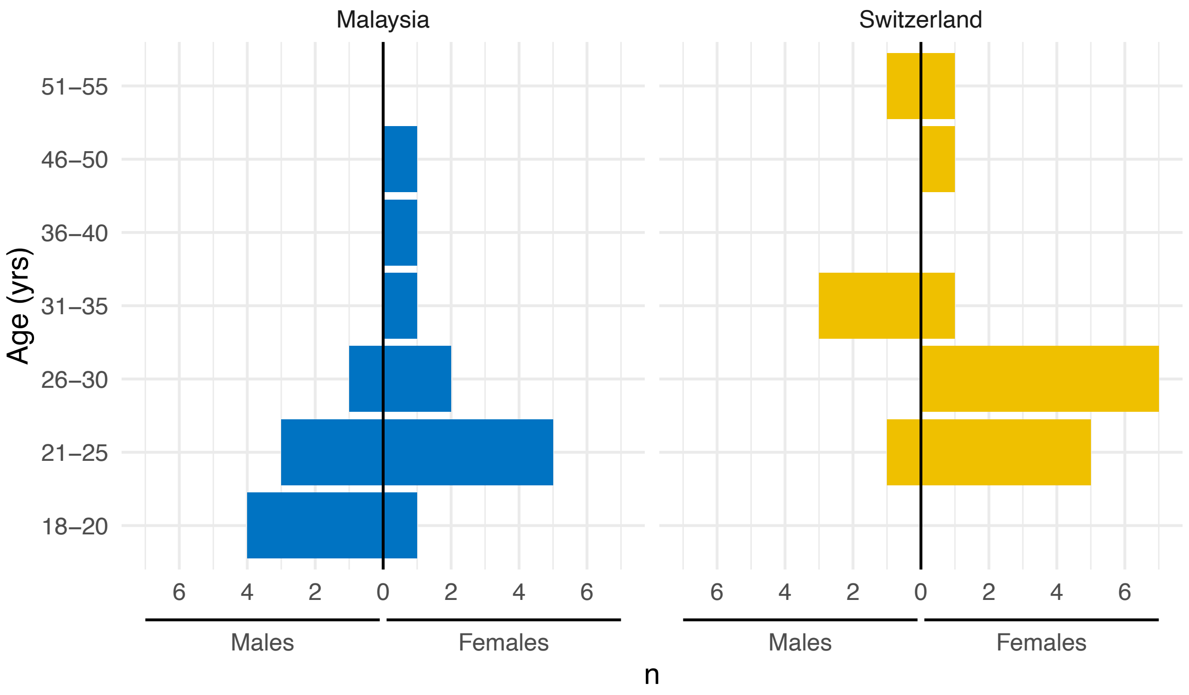

Figure S3. Distribution of age and sex between the sites. 2-way histograms for both Malaysia and Switzerland sites, showing the distribution of age in 5-year bins (+18 to 20yrs). Left of each central vertical line, male counts are shown, and females are on the right.

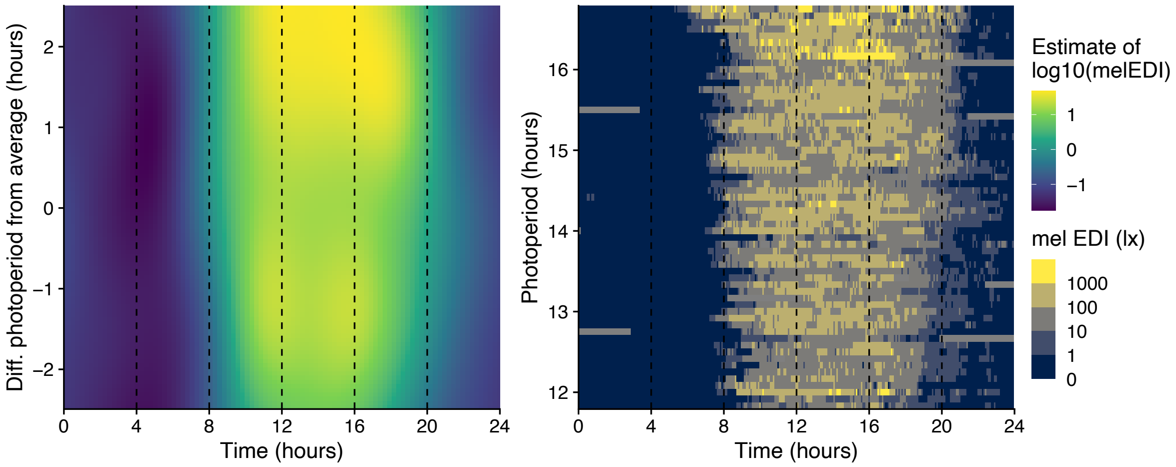

Figure S4. Light exposure patterns depending on photoperiod duration. Left: Model predictions from a generalised additive mixed-effect model of photoperiod duration, plotted as a difference in hours from the average, against the local time (in hours). Colour indicates log10 melanopic EDI. Right: Same as A, but based on measurement values, and absolute photoperiod duration. Measurements are binned into colour steps of log10 melanopic EDI.

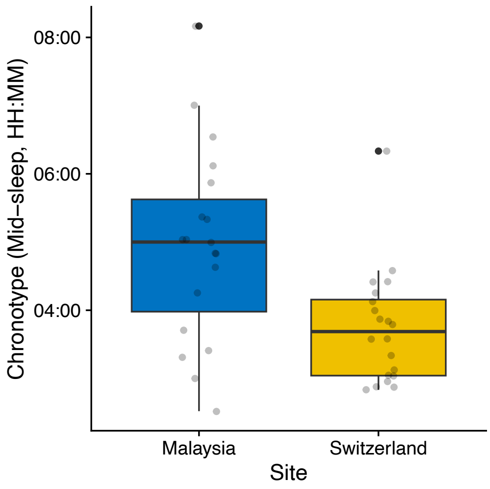

Figure S5. Chronotype distribution across sites. Boxplot with indicated individual values of chronotype by site. Chronotype is provided as the mid-sleep timing (HH:MM). Mid-sleep was 4:57 a.m. ±1.4 hours in Malaysia, and 3:45 a.m. ±0.8 hours in Switzerland. The difference is significant (*p* = *0.003*).

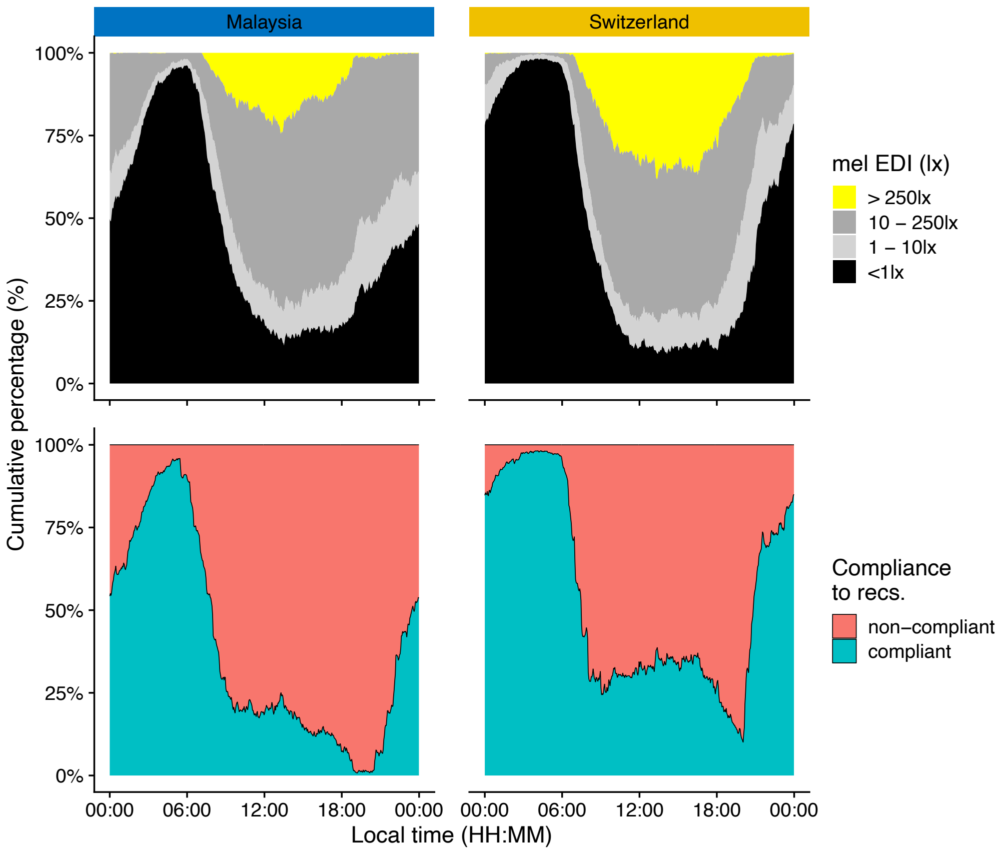

Figure S6. Light exposure in relation to the Brown recommendations for healthy lighting. Top panels: cumulative relative distribution of light exposure to recommended levels across the day; up to 1 lx mel EDI for sleep, up to 10 lx mel EDI for the evening, and above 250 lx for daytime. Bottom panels: Compliance to the recommendations across the day across all participants.

Full Analysis Document

The full analysis document can be found on our GitHub page: https://tscnlab.github.io/BillerEtAl_JExpoSciEnvironEpidemiol_2025
